# Omega-3 Fatty Acid Supplementation for 12 Weeks Increases Resting and Exercise Metabolic Rate in Healthy Community-Dwelling Older Females

**DOI:** 10.1371/journal.pone.0144828

**Published:** 2015-12-17

**Authors:** Samantha L. Logan, Lawrence L. Spriet

**Affiliations:** Department of Human Health and Nutritional Sciences, 50 Stone Road East, University of Guelph, Guelph, Ontario, N1G 2W1, Canada; National Center of Neurology and Psychiatry, JAPAN

## Abstract

Critical among the changes that occur with aging are decreases in muscle mass and metabolic rate and an increase in fat mass. These changes may predispose older adults to chronic disease and functional impairment; ultimately resulting in a decrease in the quality of life. Research has suggested that long chain omega-3 fatty acids, found predominantly in fatty fish, may assist in reducing these changes. The objective of this study was to evaluate the effect of fish oil (FO) supplementation in a cohort of healthy, community-dwelling older females on 1) metabolic rate and substrate oxidation at rest and during exercise; 2) resting blood pressure and resting and exercise heart rates; 3) body composition; 4) strength and physical function, and; 5) blood measures of insulin, glucose, c-reactive protein, and triglycerides. Twenty-four females (66 ± 1 yr) were recruited and randomly assigned to receive either 3g/d of EPA and DHA or a placebo (PL, olive oil) for 12 wk. Exercise measurements were taken before and after 12 wk of supplementation and resting metabolic measures were made before and at 6 and 12 wk of supplementation. The results demonstrated that FO supplementation significantly increased resting metabolic rate by 14%, energy expenditure during exercise by 10%, and the rate of fat oxidation during rest by 19% and during exercise by 27%. In addition, FO consumption lowered triglyceride levels by 29% and increased lean mass by 4% and functional capacity by 7%, while no changes occurred in the PL group. In conclusion, FO may be a strategy to improve age-related physical and metabolic changes in healthy older females.

***Trial Registration***: ClinicalTrials.gov NCT01734538.

## Introduction

The proportion of seniors in Canada is predicted to increase from 13% of the total population in 2005 to 25% in 2036 [1]. With age, adults experience metabolic and physical changes, including increases in heart rate (HR), blood pressure, and fat mass (FM), and decreases in resting metabolic rate (RMR), lean body mass (LM), and physical function [2, 3]. These changes predispose older adults to age-related diseases and functional impairment, ultimately resulting in an overall decrease in the quality of life (QOL). There are several strategies to maintain the health and independence of older adults, including increasing cognitive and physical activity, exercise, and optimizing nutrition [4, 5].

A family of nutrients of interest are the long-chain omega-3 fatty acids (O3FAs); specifically, eicosapentaenoic acid (EPA, C20:5n-3) and docosahexaenoic acid (DHA, C22:6n-3). Since the body can only synthesize limited amounts of EPA and DHA from alpha-linolenic acid (C18: 3n-3), these fatty acids must be obtained from the diet or through supplementation [6]. The main dietary source of EPA and DHA is seafood, with the highest concentrations found in fatty fish. The American Heart Association (AHA) and Health Canada (HC) recommend that adults consume 500 mg/d of EPA and DHA (~2 servings/wk or ~8 oz of fish/wk) [7, 8]. However, the mean intake in Western society is ~135 mg/d (~2 servings of fish/mo) [9]. Research in our laboratory also observed low intakes of O3FAs in affluent populations (~230 mg/d), despite selecting healthier foods [10].

The benefits of O3FAs are far reaching due to their integration into cell membranes. The current dietary recommendations have been developed on the premise of reducing the risk factors associated with cardiovascular disease [8, 11], with the positive health benefits often seen with doses higher (3–4 g/d) than the AHA and HC recommendations. Decreases in resting blood pressure and HR [12, 13] and improvements in the blood lipid profile (triglyceride, total cholesterol, LDL-cholesterol) [11] have been extensively researched in populations with disease or at high risk of disease.

Research has documented a decrease in RMR, and a shift in body composition towards decreased LM and increased adiposity with aging [2, 14]. Low muscle mass and a high FM are associated not only with an increased risk of many age-related disease processes, but also with mobility impairment. The decrease in RMR and LM begins around the 3^rd^ decade of life and result in declines of ~1–2%/decade for RMR [15] and ~0.26–0.56%/annum for LM [14]. The decline in RMR and LM are likely due to numerous factors, which include declining physical activity and nutrient intake, such as insufficient protein intake [16]. Skeletal muscle is responsible for ~20% of the metabolic rate at rest and up to ~80% of the energy consumption during exercise [17]. Research has suggested that O3FA intake, particularly EPA and DHA may increase RMR during rest and exercise in healthy adults, and substrate oxidation to favour a greater usage of fat [18, 19]. We recently demonstrated an increase in RMR after 12 wk of EPA and DHA supplementation and the incorporation of these fatty acids into the sarcolemmal and mitochondria membranes of human skeletal muscle of young healthy males [20, 21].

The incorporation of EPA and DHA into cell membranes may impact energy metabolism in many ways, including the regulation of cellular processes by altering gene expression, by acting as a ligand for peroxisome proliferator-activated receptors (PPARs) [22]. PPARs play an important role in energy homeostasis by regulating a wide array of genes involved in lipid metabolism [23].

Whether these benefits occur in relatively healthy (no or very low dose medications for blood pressure, cholesterol, arthritis, asthma) older adults who consume low dietary O3FAs is currently a hot topic in geriatric research. A recent study by Smith et al. [24] reported that FO supplementation (n = 40) of 4 g/d (1.86 g EPA, 1.5 g DHA) for 6 months in a cohort of healthy older adults (60–85 yr) resulted in increased: 1) thigh muscle volume by 3.6%; 2) hand grip strength by 2.3 kg, and; 3) increased 1-repetition maximum muscle strength lower and upper body strength by 4%, in comparison with a corn-oil supplemented PL group (n = 20). However, it is unknown whether changes would occur with a shorter supplementation period. For these reasons, we investigated the effect of dietary fish oil (FO) supplementation (5g/d with 2 g/d EPA and 1g/d DHA) for a 12 wk period on metabolic and physical health parameters of community-dwelling older female adults. We hypothesized that FO supplementation would result in: 1) an increased metabolic rate and a greater reliance on fat oxidation both at rest and during exercise; 2) a decrease in resting blood pressure and resting and exercise HR; 3) a decrease in adiposity and an increase in LM; 4) an increase in handgrip strength and physical function, and; 5) more healthy blood measures of high sensitivity c-reactive protein (hs-CRP) and triglycerides (TGs). A placebo (PL) group supplemented with 3 g/d of olive oil was included to control for the effects of confounding variables.

## Materials and Methods

### Recruitment and Inclusion

Twenty six females between the ages of 60–76 yr (66 ± 1) were recruited from the community of Guelph (Guelph, ON, CA). Females who met the following inclusion criteria were included in the study: (1) between the ages of 60–76 yr; (2) good cognitive status, as determined by a score >25/30 on the Mini Mental State Exam [25]; (3) consumed one meal or less of fish/wk and did not take a omega-3 supplement; (4) took no prescription medications or very low dose medications (hypertension, hypercholesterolemia, hormonal); and (5) absence of any self-reported medical diagnoses that entailed functional impairment. Following Research Ethics Board approval from the University of Guelph (S1 Protocol and S1 File), both oral and written informed consent was obtained from all participants. Consent was also attained from the participant’s medical practitioner. For the study duration, participants were instructed to maintain their current diet and physical exercise regime. Twenty four females completed the study, as two of the females dropped out prior to supplementing, due to difficulty with the time commitment and personal issues with the metabolic and blood measures (S1 CONSORT Checklist).

### Experimental Protocol

**Fig 1:**
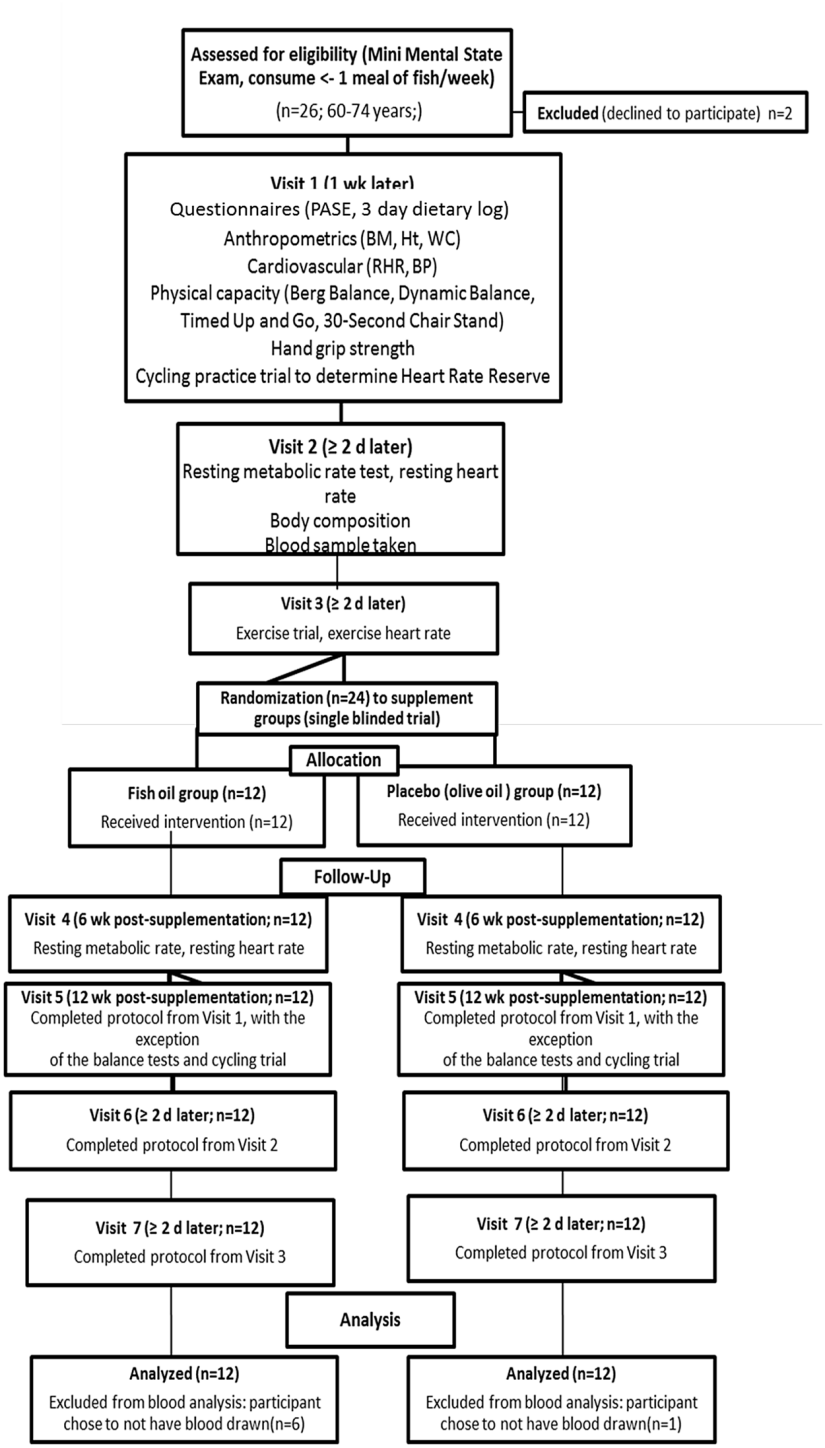
Consort figure illustrating the participant flow through the study.

After screening and recruitment, the participants reported to the laboratory on 7 separate occasions over a 14 wk period (Fig 1). Prior to all visits, participants were instructed to abstain from athletic activities and consume a mutually agreed on ‘normal’ diet [~50% energy (E) carbohydrate (CHO), ~30% E fat, and ~20% E protein] on the preceding day. During the first visit, participants completed the Physical Activity Scale for the Elderly (PASE) questionnaire, anthropometric measures (height (Ht), body mass (BM), waist circumference (WC), body composition), cardiovascular (resting HR (RHR) and blood pressure) and blood measures (fasting insulin, glucose, hs-CRP, cholesterol, TG), handgrip strength, and physical capacity measures (Berg Balance, Dynamic Index, Timed Get Up and Go (TUG), and 30-Second Chair Stand (30-SCS)). Participants also completed a cycling practice trial on an electronically braked cycle ergometer (LODE Excalibur; Quinton Instrument, Groningen, The Netherlands) to determine the power output needed to maintain the participant’s HR within a zone of low intensity (40% of HR reserve, HRR). The HRR was calculated as HRR = (maximal HR − resting HR) + resting HR [26]. The participants also evaluated their cycling intensity during all exercise trials using the Rating of Perceived Exertion (RPE) Scale [27].

During the second visit, participants reported to the laboratory following a 12-hr overnight fast and were instructed to lay supine in a darkened room for 30 min. Participants provided breath samples during the last 15 min to measure RMR. The volume of oxygen consumed (VO_2_; mL/min) and carbon dioxide produced (VCO_2_; mL/min) were determined using a metabolic cart (MOXUS metabolic system; AEI Technologies, Pittsburgh, PA, US). HR was recorded every 5 min with a heart rate monitor (Polar Electro, Inc., Port Washington, NY, US). After the RMR measurement, body composition was analyzed for FM and fat-free mass or LM using bioelectrical impedance analysis (Bodystat 1500, FL, US), and a resting venous blood sample was taken.

At least 2 days later, participants reported to the laboratory for a third visit to complete a 30 min exercise trial. Participants were instructed to eat a mutually agreed upon breakfast (~50% E CHO, ~30% E fat, and ~20% E protein (~350 kcal)) 2 hr before arriving to the laboratory and drink 500 mL of water within the 2 hr before arrival to ensure hydration. The participant was asked to provide a detailed log of their breakfast and instructed to consume the same breakfast on the post supplementation testing day. The participants then completed 30 min of low intensity cycling exercise at the power output established from the first visit. HR was recorded every 5 min and 4 min respiratory gas measurements were collected at the end of every 10 min (6–10, 16–20, 26–30 min). Following the third visit, the participants were matched by age, body mass index (BMI), and medication use, and were randomly assigned in a single-blinded manner to one of two supplement groups: fish oil (FO, 12 females) or placebo (PL, 12 females). The FO group took 5 g/d of FO (Omega-3 Complete, Jamieson Laboratories Ltd., Windsor, ON, CA) administered in 5 capsules, with each capsule providing 400 mg of EPA and 200 mg of DHA (daily total, 2 g EPA and 1 g DHA). The PL group took 3 g/d of olive oil (Swanson EFAs, Certified Organic Olive Oil, Swanson Health Products, Fargo, ND, US) administered in 3 capsules. To reduce any minor side-effects of the oils (burping, indigestion), the participants were instructed to take the supplements frozen and with meals; with the FO group taking 1 capsule at breakfast, 2 at lunch, and 2 at supper; and the PL group taking 1 capsule at each meal. To encourage compliance, the first month of supplements were provided in daily packets. After 4 wk the capsules were allotted in weekly amounts. In addition to picking up the supplements, participant compliance was encouraged with periodic phone calls and email reminders. Following 6 wk of supplementation, participants completed the 12 hr overnight fasted and resting protocol from the second lab visit, without the blood sample. After 12 wk of supplementation, the participants repeated the protocol from visits 2, 3, and 4, with the exception of the balance measures from visit 2. Dietary and physical activity compliance were assessed by monitoring records at the start and end of the study. Dietary intake was assessed by the completion of a three day food record and physical activity by the PASE questionnaire pre and post supplementation [28].

At the end of the study the participants were asked which supplement group they believed they were in. The majority of the participants taking FO (65%) and PL (55%) correctly identified their supplement group. The only supplementation symptoms reported were belching and heartburn, as 70% of the FO participants reported belching on ~1–2 d/wk and 10% reported heartburn on ~3–4 d/wk. Overall, the PL supplement was well tolerated, with only one participant reporting heartburn on ~3–4 occasions/wk during the last 6 wk of supplementation.

### Physical Measures

All body composition (Ht, BM, WC) and grip strength measures were conducted as outlined in the Canadian Physical Activity, Fitness and Lifestyle Approach (CPAFLA) [29]. Briefly, Ht was measured to the nearest 0.1 cm using a vertical metric wall tape and a horizontal flat edge, BM was measured to the nearest 0.1 kg on a calibrated digital scale (Health O Meter; Bridgeview, IL, US) and WC was measured to the nearest 0.5 cm, and was taken at the top of the iliac crests using an anthropometric tape. BMI was calculated as BM/Ht^2^.

Bioelectrical Impedance Analysis (Bodystat 1500, FL, US) was completed directly after the 12-hr fasted and RMR measures, where the participant continued to lay supine with limbs abducted, and leads were attached according to manufacturer’s instructions. Fat free mass index (FFMI) was calculated using LM and standardizing for height (LM (kg)/ Ht^2^ (m^2^)) [30]. Isometric handgrip strength was measured using a hydraulic hand-held dynamometer (Vernier Jamar; Sammons Preston Rolyan; Nottinghamshire, ENG, UK). Three measurements per hand were taken and the participant alternated hands between measurements to allow ~30 s of rest. The highest measurement for each hand was added to achieve the combined grip strength (CGS) value.

To assess functional capacity, the Berg Balance, Dynamic Gait Index, TUG, and the 30–30-SCS tests were employed as described elsewhere [30–32]. Since the functional capacity, as determined by the Berg Balance and Dynamic Index tests, of the cohort was high, we only repeated the TUG test and the 30-SCS post-supplementation.

### Cardiovascular and Blood Measures

Resting systolic and diastolic blood pressures (SBP, DBP; mmHg) were measured using a blood pressure monitor (OMRON IntelliSense; Model HEM-907XL; OMRON Healthcare, IL, US). Participants were seated with their left arm resting on a table for 3 min prior to three blood pressure measurements taken 1 min apart. There was no significant difference between the three resting values so the mean of all measurements were used for data analysis.

After a 12 hr overnight fast, venous blood was collected and analyzed for serum glucose, insulin, hs-CRP, fatty acids, and TGs (mmol/L). The bloods were analyzed at LifeLabs Medical Laboratory Services (Guelph, ON, CA)

### Metabolic Calculations

For both the rest and exercise trials, the VO_2_ and VCO_2_ were measured and used to calculate the respiratory exchange ratio (VCO_2_/VO_2_, RER) and whole body carbohydrate oxidation (CHO Ox) and fat oxidation (Fat Ox) by using the non-protein RER table and the following equations:

CHO Ox (g) = (4.585 x VCO2) − (3.226 x VO2);

and

Fat Ox (g)= (1.695 x VO2)− (1.701 x VCO2).

The RMR or energy expenditure (Energy Ex) was calculated using the thermal equivalent of VO_2_ consumed based on non-protein respiratory quotient table and the following equation [33]:

Energy Ex (Kcal) = VO2 (L/min) x RER cal equiv (Kcal/L) x Time (min).

### Questionnaires

The participants completed the PASE questionnaire, designed to measure the amount of physical activity engaged in over the past 7 d, with higher scores indicative of greater amounts of daily activity [28].

### Assessment of Dietary Intake

The participants were asked to record their food and beverage consumption using a multiple-day food record (version 3; Fred Hutchison, WA, US) on 3 consecutive days, which included 2 weekdays and 1 weekend day. Detailed instructions were provided to the participants to ensure accurate dietary intake recording. The dietary information was entered into the Food Processor SQL-ESHA database version 10.8.0 (ESHA Research, Salem, OR, US).

### Statistical Analysis

After determining data normality and variance homogeneity using the Shapiro-Wilk test and Levene’s Test for Equality of Variances, a 2-way analysis of variance (ANOVA, time and groups) was used to determine if interactions between time and groups existed for the physical and metabolic measures. When a significant F-ratio was obtained, Tukey’s post hoc tests were used to determine where the significance occurred. Data are presented as means ± SE. Statistical significance was accepted as *p*<0.05 for all tests. Statistics were computed using PASW Statistics 19.0.1 for Windows (Chicago, IL, US).

## Results

### Participant Characteristics

The participant data indicated that the cohort was generally in good health, although the body composition data denoted that the cohort was overweight (Table 1). The mean FFMI values indicated that the participants had healthy amounts of skeletal muscle. The cohort was also high functioning according to the balance, TUG, and 30-SCS tests, and possessed healthy CGS values (Table 1). The physical activity level of the cohort (PASE score) was above average in comparison to sex and age matched normative data for older adults [28]. Further, the nutrition data demonstrated that the total energy intake for the females was indicative of a low to moderate level of daily activity, and low levels of daily activity [34]. The participants also consumed healthy amounts of fat, CHO and protein [35], and the average energy intake did not significantly change over the supplementation period. Compliance was demonstrated by the significant increase in EPA (0 wk, 0.86 ± 0.10; 12 wk, 5.97 ± 0.62) and DHA (0 wk, 1.77 ± 0.09; 12 wk, 3.79 ± 0.19) in the FO group, while serum levels of EPA (0 wk, 0.85 ± 0.13; 12 wk, 1.04 ± 0.13) and DHA (0 wk, 1.73 ± 0.22; 12 wk, 1.92 ± 0.27) in the PL group did not change. The cardiovascular data indicated that the mean SBP and DBP were in the healthy range (Table 2). Further, the fasted blood data indicated that all of the participants had healthy insulin, glucose, and TG values. The hs-CRP and TC values indicated that a low level of risk of cardiovascular disease was evident (Table 1). Finally, the use of medication was low in the cohort, with 42% taking any form of low-dose medications.

**Table 1:** Participant health and physical measures at 0 and 12 wk of supplementation with placebo or fish oil.

	Placebo (n = 12)		Fish Oil (n = 12)	
	0 Wk	12 Wk	0 Wk	12 Wk
**Body Composition & Cardiovascular**				
**Body Mass (kg)**	69.1 ± 3.0	69.0 ± 3.1	72.9 ± 3.0	73.3 ± 3.7
**Body Mass Index (kg/m** ^**2**^ **)**	26.3 ± 1.0	26.3 ± 1.1	27.9 ± 1.3	28.0 ± 1.2
**Waist Circumference (cm)**	91.4 ± 3.1	90.2 ± 2.7	92.6 ± 2.3	92.1 ± 2.9
**Fat Mass (kg)**	29.6 ± 1.9	28.9 ± 2.0	32.6 ± 2.0	31.3 ± 2.2
**Lean Mass (kg)**	39.5 ± 1.4	40.1 ± 1.6	40.3 ± 1.2	41.9 ± 1.3 *
**Systolic Blood Pressure (mmHg)**	119 ± 3.3	116 ± 4.9	117 ± 4.8	115 ± 3.8
**Diastolic Blood Pressure (mmHg)**	72 ± 1.9	72 ± 2.6	70 ± 3.5	66 ± 2.6
**Function & Strength**				
**Combined Grip Strength (kg)**	57.4 ± 2.3	57.1 ± 2.4	49.9 ± 2.8	51.5 ± 3.4
**Timed Up and Go Test (s)**	7.3 ± 0.2	7.1 ± 0.2	7.6 ± 0.2	7.1 ± 0.2 *
**30-Second Sit To Stand (# Completed)**	15 ± 0.9	17 ± 1.1	15 ± 1.7	17 ± 1.9
**PASE**	120 ± 21	124 ± 22	149 ±15	152 ± 18
**Dietary Intake**				
**Total Energy Intake (kcal)**	1926 ± 166	2009 ± 155	1867 ± 107	1924 ± 140
**Fat (g)**	64 ± 6	71 ± 9	59 ± 6	64 ± 6
**Protein (g)**	72 ± 5	81 ± 6	81 ± 5	86 ± 4
**Carbohydrate (g)**	258 ± 28	261 ± 17	253 ± 25	269 ± 21
**Fasted Blood**	**n = 6**		**n = 11**	
**Insulin (pmol/L)**	65.6 ± 21.3	61.2 ± 15.8	52.1 ± 4.4	49.4 ± 6.6
**Glucose (mmol/L)**	5.0 ± 0.3	4.96 ± 0.26	5.04 ± 0.13	5.12 ± 0.13
**C-Reactive Protein (mg/L)**	1.75 ± 0.33	1.67 ± 0.25	3.28 ± 0.70 ^+^	3.29 ± 0.59 ^++^
**Triglycerides (mmol/L)**	1.19 ± 0.15	1.13 ± 0.13	1.30 ± 0.14	1.01 ± 0.14 *
	**n = 6**		**n = 9**	
**EPA (% of total fatty acids)**	0.85 ± 0.13	1.04 ± 0.13	0.86 ± 0.10	5.97 ± 0.62 *
**DHA (% of total fatty acids)**	1.73 ± 0.22	1.92 ± 0.27	1.77 ± 0.09	3.79 ± 0.19 *
**EPA (absolute %)**	14.50 ± 2.40	19.71 ± 2.49	13.85 ± 1.82	88.17 ± 9.34 *
**DHA (absolute %)**	28.99 ± 3.89	36.48 ± 5.52	27.73 ± 1.66	57.47 ± 3.96 *

Data are means (±SE). PASE = physical activity score for the elderly questionnaire; EPA = eicosapentaenoic acid; DHA = dicosahexaenoic acid. Significant difference *within groups at 0 and 12 wk, and between groups at ^+^0 wk and ^++^12 wk.

**Table 2:** Resting metabolic measures at pre, 6 wk and 12 wk post supplementation with placebo or fish oil.

Measure	Placebo (n = 12)			Fish Oil (n = 12)		
	0 Wk	6 Wk	12 Wk	0 Wk	6 Wk	12 Wk
**VO**_**2**_ **(mL/min)**	179.9 ± 7.6	180.2 ± 8.0	183.0 ± 7.6	169.4 ± 6.8	189.5 ± 7.1 *	197.0 ± 7.4 **
**VCO**_**2**_ **(mL/min)**	132.4 ± 5.8	134.0 ± 4.9	134.1 ± 5.0	129.3 ± 6.8	144.8 ± 7.4	147.7 ± 7.0 **
**Respiratory Exchange Ratio (RER)**	0.73 ± 0.02	0.74 ± 0.02	0.73 ± 0.02	0.76 ± 0.01	0.76 ± 0.01	0.75 ± 0.02
**Resting Heart Rate (bpm)**	65 ± 2	66 ± 2	66 ± 2	63 ± 2	60 ± 2 *	60 ± 2 ** ^++^
**Rate of Fat Oxidation (mg/min)**	79.7 ± 6.6	77.5 ± 7.1	82.2 ± 7.5	67.2 ± 6.3	74.9 ± 6.4	82.7 ± 6.6 **
**Rate of Carbohydrate Oxidation (mg/min)**	26.7 ± 12.5	33.1 ± 11.6	24.4 ± 11.8	46.3 ± 11.7	52.6 ± 13.5	41.7 ±12.6
**RMR (kcal/min)**	0.85 ± 0.04	0.85 ± 0.06	0.86 ± 0.09	0.80 ± 0.05	0.90 ± 0.04 *	0.93 ± 0.04 ** ^++^
**RMR Normalized for Body mass (kcal/kg)**	0.18 ± 0.01	N/A	0.19 ± 0.02	0.17 ± 0.01	N/A	0.19 ± 0.01 **
**RMR Normalized for Lean Mass (kcal/kg)**	0.33 ± 0.02	N/A	0.32 ± 0.03	0.30 ± 0.01	N/A	0.33 ± 0.01 **

Data are means (±SE). RMR = resting metabolic rate. The data in the table are the last 15 min of a 30 min rest period. Significant difference within groups *at 0 and 6 wk, **at 0 and 12 wk, and between groups at ^++^12 wk.

The groups were well matched for the baseline physical and metabolic measures (*p*>0.05), with the exception of hs-CRP values, where the FO group had significantly higher baseline values of hs-CRP than the PL group (Table 2).

### Influence of Supplementation on Physical and Blood Measures

After 12 wk of supplementation, a significant increase (4%; *p* = 0.01) in LM of 1.6 ± 0.7 kg and decrease in TUG speed (7%; *p* = 0.006) of 0.5 ± 0.2 s was found in the FO group, while no significant changes were found in the PL group for LM or TUG. TG values also significantly decreased (29%, *p* = 0.001) by 0.29 ± 0.07 mmol/L for the FO females, while no significant change was found in the PL group (Table 1). Finally, there were no significant changes in any of the other body composition, physical function and strength measures (Table 1), or in the other cardiovascular and blood values for both the FO and PL groups over time (Table 2).

### Influence of Supplementation on Resting Metabolic Measures

**Fig 2:**
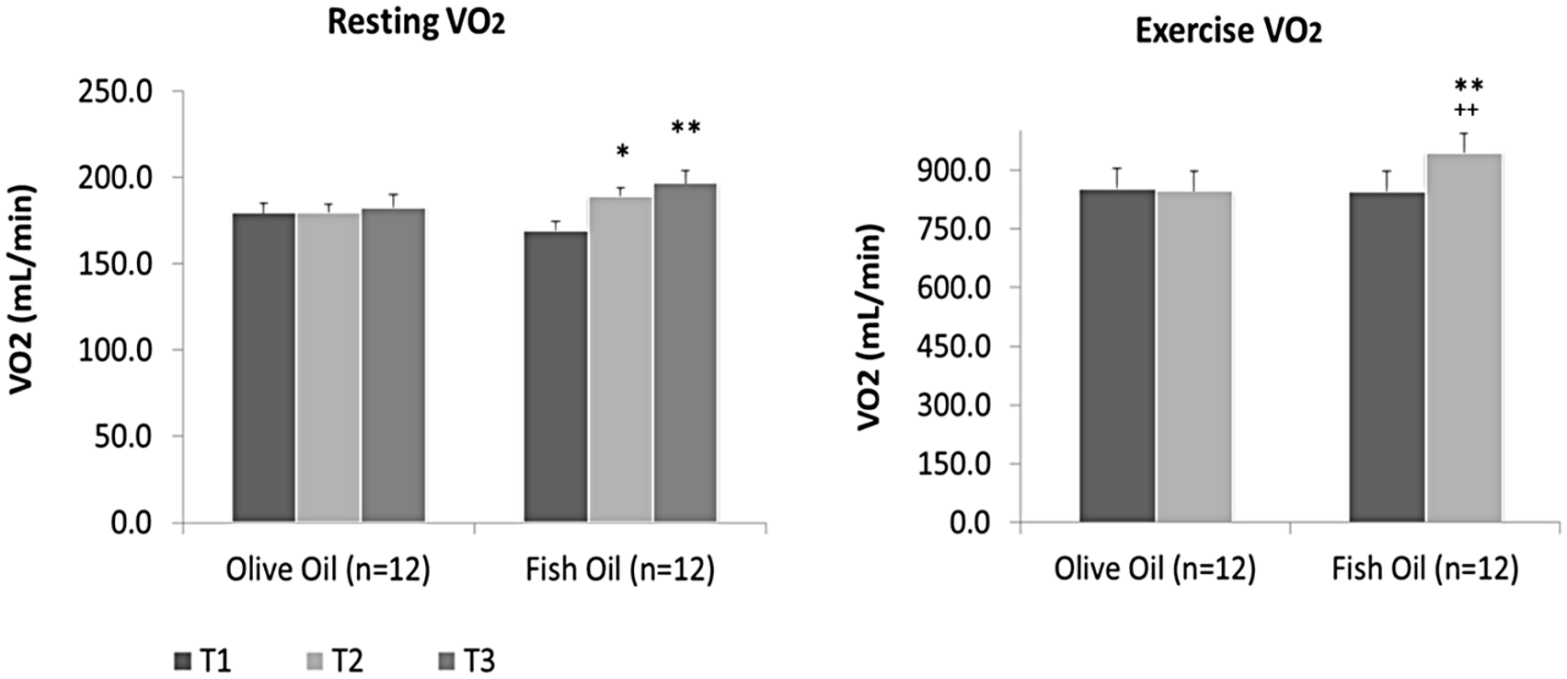
Oxygen uptake measures at rest and during exercise at baseline (time 1 (T1); 0 wk), and after 6 (T2) and 12 wk (T3) of placebo (olive oil) or fish oil supplementation. Significant difference within groups *at 0 and 6 wk (*p* = 0.003), **at 0 and 12 wk (Resting *p* = 0.003; Exercise *p* = 0.002), and between groups ^++^ at 12 wk (*p* = 0.004).

**Fig 3:**
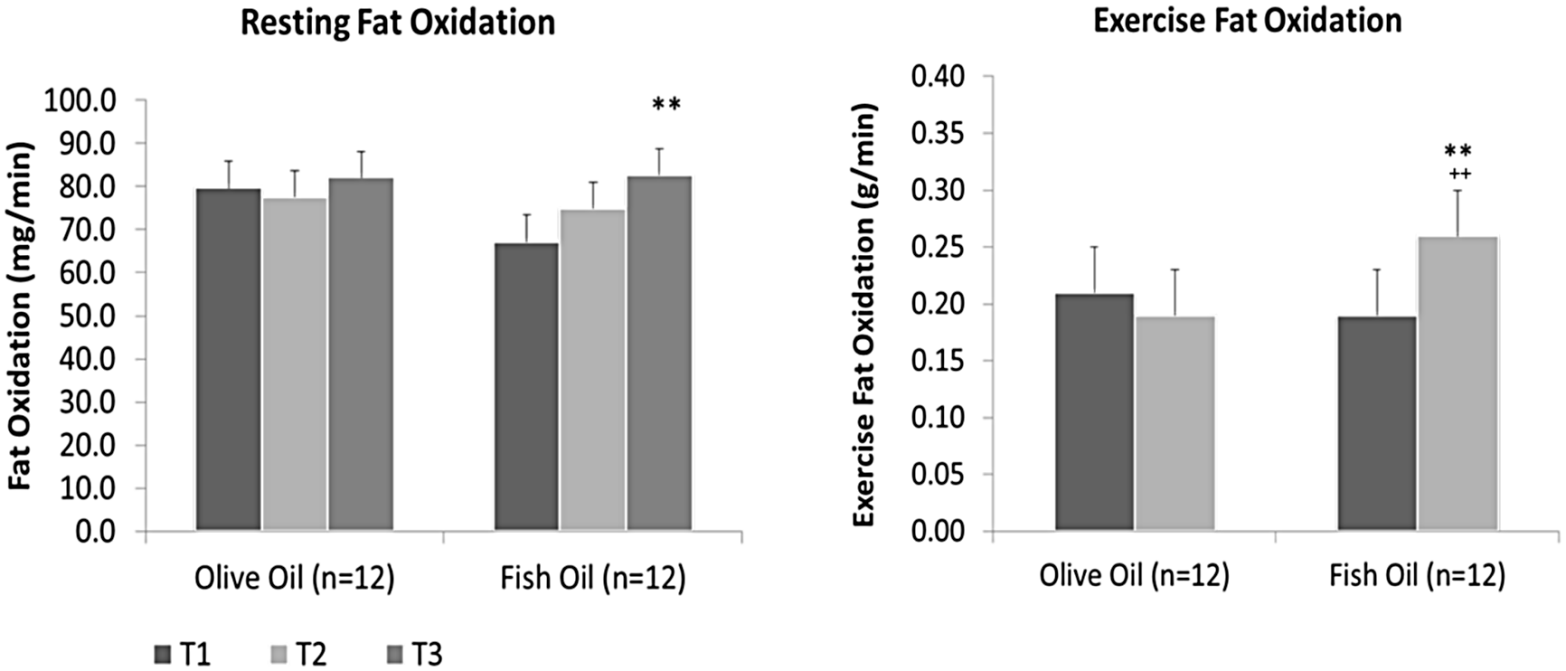
Metabolic measures of energy expenditure at rest and during exercise at baseline (time 1 (T1); 0 wk), and after 6 (T2) and 12 wk (T3) of placebo (olive oil) or fish oil supplementation. Significant difference within groups *at 0 and 6 wk (*p* = 0.003), **at 0 and 12 wk (Resting *p* = 0.003; Exercise *p* = 0.006), and between groups at ^++^12 wk (Resting *p* = 0.003; Exercise *p* = 0.004).

Significant increases in VO_2_ of 20.1 ± 8.9 mL/min after 6 wk (11%; *p* = 0.003), and 27.6 ± 8.0 mL/min after 12 wk (14%; *p* = 0.003) of supplementation occurred in response to FO intake (Table 2, Fig 2), while no significant changes occurred in the PL group. Resting VCO_2_ followed a similar trend, with a significant increase (12%; *p* = 0.004) in the FO group of 18.4 ± 5.73 mL/min after 12 wk of supplementation while no changes were found in the PL group (Table 2). The changes in VO_2_ resulted in a significant increase in RMR of 0.10 ± 0.04 kcal/min at 6 wk (11%; *p* = 0.003) and 0.13 ± 0.04 kcal/min after 12 wk (14%; *p* = 0.003) of FO supplementation (Table 2, Fig 3). No changes in RMR were evident in the PL group. Finally, RMR remained significantly increased in the FO group when normalized for BM (11%; *p* = 0.005) and LM (9%; *p* = 0.004) (Table 2, Fig 3).

**Fig 4:**
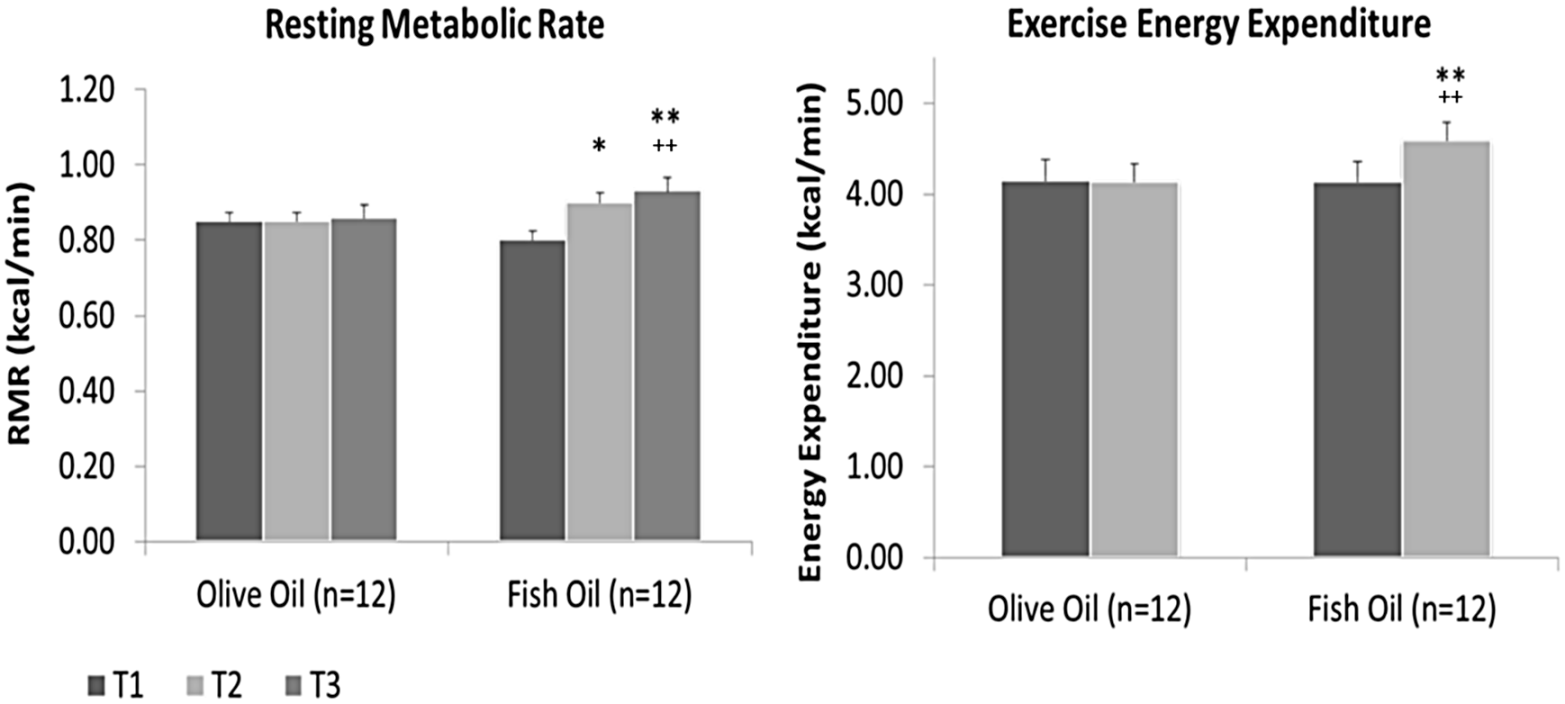
Fat oxidation at rest and during exercise at baseline (time 1 (T1); 0 wk), and after 6 (T2) and 12 wk (T3) of placebo (olive oil) or fish oil supplementation. Significant difference within groups at ** 0 and 12 wk (Resting *p* = 0.003; Exercise *p* = 0.002), and between groups at ^++^12 wk (*p* = 0.004).

The substrate oxidation data indicated that FO supplementation resulted in a significant increase (19%; *p* = 0.003) in the rate of Fat Ox of 15.5 ± 5.7 mg/min, while no significant changes were found in the PL group after 12 wk of supplementation (Table 2, Fig 4). Further, no significant changes were found in the rate of CHO Ox for the FO and PL groups. Finally, FO supplementation significantly decreased RHR by 3 ± 1 bpm after 6 wk (5%; *p* = 0.021) without further decreases at 12 wk. RHR remained significantly unchanged in the PL group (Table 2).

### Influence of Supplementation on Exercise Metabolic Measures

The average power output during the exercise trial was 36 ± 4 W for the PL group and 35 ± 6 W for the FO group. VO_2_ significantly increased (10%; *p* = 0.002) by 99.2 ± 19.7 mL/min for the FO group after 12 wk of supplementation, while no changes were evident in the PL group (Table 3, Fig 2). Exercise VCO_2_ followed a similar trend where a significant increases (8%; *p* = 0.004) of 60.7 ± 20.1 mL/min occurred in response to FO supplementation at 12 weeks while no changes were found in the PL group (Table 3). RER was unchanged in the FO and PL groups. Total Energy Ex increased significantly (10%; *p* = 0.006) by 13.8 ± 2.5 kcal in the FO group only (Table 3, Fig 3). When expressed as a rate, an increase of 0.46 ± 0.08 kcal/min (10%, *p* = 0.006) for the FO group occurred, with no significant changes PL group. Finally, Energy Ex remained significantly increased when normalized for BM (11%; *p* = 0.009) and LM (6%; *p* = 0.011) (Table 3).

**Table 3:** Exercise metabolic measures pre and post 12 wk supplementation with placebo or fish oil.

Measure	Placebo (n = 12)		Fish Oil (n = 12)	
	0 Wk	12 Wk	0 Wk	12 Wk
**VO**_**2**_ **(mL/min)**	854.1 ± 50.1	847.5 ± 54.8	846.4 ± 43.9	945.6 ± 42.4 * ^++^
**VCO**_**2**_ **(mL/min)**	728.9 ± 41.2	730.8 ± 41.1	731.0 ± 34.2	791.7 ± 36.2 * ^++^
**Respiratory Exchange Ratio (RER)**	0.85 ± 0.02	0.86 ± 0.02	0.86 ± 0.01	0.84 ± 0.02
**Heart Rate (bpm)**	103 ± 3	101 ± 3	106 ± 1	104 ± 1 *
**Total Fat Oxidation (g)**	6.2 ± 1.3	5.8 ± 1.6	5.8 ± 1.1	7.7 ± 1.2 * ^++^
**Rate of Fat Oxidation (g/min)**	0.21 ± 0.04	0.19 ± 0.05	0.19 ± 0.04	0.26 ± 0.04 * ^++^
**Total Carbohydrate Oxidation (g)**	17.6 ± 2.5	18.5 ± 2.4	18.6 ± 2.1	17.4 ± 2.0
**Rate of Carbohydrate Oxidation (g/min)**	0.59 ± 0.08	0.62 ± 0.08	0.62 ± 0.07	0.58 ± 0.07
**Total Energy Ex (kcal)**	124.6 ± 7.0	123.9 ± 7.1	123.8 ± 6.3	137.6 ± 6.5 * ^++^
**Rate of Energy Ex (kcal/min)**	4.15 ± 0.23	4.13 ± 0.24	4.13 ± 0.21	4.59 ± 0.22 * ^++^
**Energy Ex Normalized for Body mass (kcal/kg)**	1.8 ± 0.1	1.8 ± 0.1	1.7 ± 0.1	1.9 ± 0.1 *
**Energy Ex Normalized for Lean Mass (kcal/kg)**	3.2 ± 0.2	3.1 ± 0.2	3.1 ± 0.2	3.3 ± 0.2 * ^++^

Data are means (±SE) of a 30 min exercise trial. The average power output (W) was 36 (±4) for PL females, and 35 (±6) for FO females. Significant difference *within groups at 0 and 12 wk, and between groups at ^++^12 wk. Ex = expenditure.

FO supplementation significantly increased (25%; *p* = 0.002) total Fat Ox by 1.9 ± 0.6 g (Table 3). When calculated as a rate, an increase (27%; *p* = 0.002) of 0.07 ± 0.02 g/min for the FO group occurred (Fig 4), while no significant changes were found in the PL group (Table 3). Further, no significant changes were found in total CHO Ox or in the rate of CHO Ox over 12 wk of FO and PL supplementation.

Finally, exercise HR was significantly decreased (2%; *p* = 0.02) by 2 ± 1 bpm in response to FO supplementation (Table 3), with no significant changes in the PL group.

## Discussion

This study demonstrated that FO supplementation (2 g EPA, 1 g DHA/d) for 12 wk in community-dwelling older female adults resulted in: 1) increased metabolic rate and fat oxidation both at rest and during exercise; 2) decreased resting and exercise HR; 3) increased LM and physical function, and; 4) decreased fasted blood TGs. In addition, supplementation with a PL (3 g olive oil/d) had no effect on any measures.

### Influence of Supplementation on Metabolism

To our knowledge, this is the first study to evaluate and demonstrate an increase in resting and exercise metabolic rates after 12 wk of FO supplementation in healthy older females. The increase in RMR following FO supplementation was impressive after 12 wk of supplementation (14%), with the majority of the significant changes present after 6 wk (11%). Extrapolated to a 24 hr period, this translates into an increased expenditure of ~187 kcal/d. Further, we observed an increase in resting FAT Ox of 19% without a significant change in resting CHO Ox. Similar results occurred in response to the exercise challenge as Energy Ex increased significantly after 12 wk of supplementation (10%) and Fat Ox increased by 27%, with no significant change in CHO Ox.

Conflicting results are present in the literature regarding the influence of FO intake on resting and exercise oxygen consumption and substrate metabolism in humans. In fact, the majority of previous research has been conducted in younger humans and has not demonstrated significant changes with O3FA supplementation [20, 36, 37]. In a previous study from our laboratory, we reported small and variable increases in RMR in young men following FO supplementation, but no changes in substrate oxidation [20]. However, significant increases in FAT Ox have been reported in response to O3FA intake [18, 36]. Couet et al. [18] reported a significant increase in FAT Ox (22%), similar to our resting data, and a decrease in FM (0.9 kg) when 6 g/d of fat in the diet was replaced with FO (1.1 g/d EPA, 0.7 g/d DHA) for 3 wk in young adults (n = 5 males, 1 female, 23 ± 2 yr). They also reported an increase in RMR, but when the increase in LM was accounted for, the RMR increase was not significant; suggesting that the FO may increase RMR by increasing LM [18]. This is contrary to our results where the increase in Energy Ex at rest and during exercise remained significant when normalized for BM and LM (Tables 2 and 3), suggesting that changes in LM were not the main factor influencing the increase in energy expenditure. Although the tissues predominantly involved in the increase in metabolic rate in this study are unknown, skeletal muscle is likely to be involved. Skeletal muscle comprises ~20% of energy expenditure at rest and increases to ~80% of oxygen use during exercise [17]. However, while other metabolically active tissues (heart, lungs, kidneys, brain and liver, etc.) may be contributing to this increase in metabolic rate at rest, during exercise we observed a greater absolute increase in energy expenditure, suggesting a large role for skeletal muscle [38].

The mechanisms by which EPA and DHA modulate energy metabolism are speculated to be due to their ability to activate and bind various PPAR isoforms [39]. By activating PPARs, changes in energy metabolism may result by influencing mRNA, protein expression, and the activity of various proteins. Proposed changes with EPA and DHA intake include an increase in 1) mRNA expression of fatty acid translocase/Cluster of Differentiation 36 (FAT/CD36), a transport protein involved in the movement of fatty acids across the sarcolemmal and mitochondrial membranes [40]; 2) Fatty acid-binding protein (FABPc), an intracellular transport protein that chaperones fatty acids in the cytoplasm for storage or transport into the mitochonria for oxidation [41]; 3) mRNA of uncoupling protein-3 (UPC3), a transport protein associated with the flow of anions from the inner to the outer mitochondrial membrane and the return transfer of protons [20, 42, 43]; 4) mRNA expression of peroxisomal acyl-CoA oxidase, an enzyme that catalyzes fatty acid oxidation [42], and; 5) an increase in carnitine palmitoyltransferase I (CPTI) activity, a rate-limiting enzyme in fatty acid oxidation [44]. Finally, O3FAs may also affect energy metabolism through up-regulation of peroxisome proliferator-activated receptor gamma coactivator 1-alpha (PGC-1α), a transcriptional coactivator that is involved in regulating the genes involved in energy metabolism and in mitochondrial biogenesis and function [45, 46].

Much remains unknown regarding how O3FAs influence oxygen consumption and energy expenditure by incorporation into the membrane. Several theories have been proposed to explain the mechanisms of O3FAs. The pacemaker theory proposes that an increase in membrane unsaturation is associated with an elevated metabolic rate by increasing membrane proteins or membrane associated processes. In fact, elevated DHA content in cell membranes has been associated with higher amounts of Ca^2+-^ATPase and Na^+^/K^+^-ATPase proteins which use adenosine triphosphate (ATP) on a continual basis [47], resulting in an increased ATP consumption and thus a higher metabolic rate [48]. This will need to be examined in future studies with skeletal muscle biopsies in older females.

A second theory suggests that the incorporation of O3FAs into mitochondrial membranes results in an increased oxygen consumption and energy expenditure. This is speculated to occur due to a proton leak across the inner mitochondrial membrane via increased UCP3 protein content, which reduces the energy coupled to ATP production and ultimately results in a reduced energy yield [48]. This did not appear to be the case in a study with FO supplementation in young males, as state 4 mitochondrial respiration was unaffected and reactive oxygen species were higher in isolated and permeabilized skeletal muscle fibers [21]. Again, this will need to be tested in skeletal muscle of older populations.

### Influence of Supplementation on Physical Measures

The potential of O3FAs to maintain or increase LM is of interest, since beginning around the 3^rd^ decade of life adults experience an annual decline in muscle mass of 0.26–0.56% which may result in decreased metabolic and physical health [14]. Because O3FA intake has been speculated to increase post-prandial satiety, which may decrease caloric intake and lead to a decreased BM [49], we ensured that dietary intake and physical activity (PASE score) remained unchanged during the study period (Table 1). To our knowledge this is the first study to report an increase in LM after supplementing with 6 wk of fish oil in healthy older females. Our results demonstrated a significant increase in LM by 4% for the FO females without a significant change in BM. This suggests that 12 wk of FO intake may ameliorate the age-associated decreases in lean mass. However, the use of a more precise measurement of body composition, such as dual x-ray absorptiometry (DEXA) or magnetic resonance imaging, is needed to detail where this increase in LM is occurring. Recently, Smith et al. [24] reported a similar increase (3.5%) in thigh muscle LM using DEXA. Our studies differ in that the Smith et al. subjects were supplemented with a lesser daily amount of EPA (1.86 g) and DHA (1.5 g), but for a longer period (6 months) in comparison to the present study (3g EPA, 2g DHA; 3 months). In a previous study, this group also reported an increase in muscle protein synthesis in response to 8 wk of supplementation with 1.9 g/d EPA and 1.5 g/d DHA in healthy older adults (10 males, 6 females; 71 ± 1 yr) [50]. Further, it was demonstrated that 6 wk of FO supplementation (1.6 g/d of EPA and 0.8 g/d of DHA) in a healthy younger adult population (6 males and 16 females; 33 ± 13 yr, mean + SD) can result in a decreased FM (-0.5 ± 1.3 kg) and an increased LM (+0.5 ± 0.5 kg) without a change in BM [19]. Thus, a dose response study appears warranted to determine the amount and time required to increase LM, and whether or not a plateau occurs at a certain dose or after a certain time period.

Very little research has been conducted regarding the role of FO supplementation on strength and physical function. Although we did not observe significant increases in combined grip strength, the FO females did experience a small but significant (7%) increase in physical function, as determined by TUG speed (0.5 ± 0.2 s) (Table 1). This is the first study to report a small but significant effect of FO supplementation and TUG speed in healthy older adults independent of a physical training protocol. Previous research reported an increase in physical performance, as measured by walking speed, in response to supplementation with 1.2 g/d of EPA and DHA for 6 months in frail older women (n = 126; 75 ± 6 yr, mean + SD) [51]. Other research in older women (n = 45, 64 ± 1.4 yr) reported that FO supplementation (2 g/d), in addition to a 90 d strength training protocol, resulted in significant increases in functional capacity (sit to stand test, but not walking speed), muscular strength, and neural activation (as measured by electromyography), which were greater than values achieved by strength training alone [52]. Recently, Smith et al. [24] reported an increase in handgrip strength by 2.3 kg, and an increase in 1-repetition maximum strength by 4% in response to FO supplementation (EPA: 1.86 g; DHA: 1.5 g) for a 24 week period. It may be that the supplementation period of the current study was not long enough to evoke increases in strength. Thus, future research should aim to determine the length of supplementation required to elicit significant changes. Although the mechanisms to explain the increase in physical function are currently unknown, it is speculated that O3FAs may improve muscular function by increasing both the fluidity of the membrane and acetylcholine sensitivity [53]. At the neuromuscular junction, acetylcholine assists in muscle contraction by facilitating fast synaptic transmission, resulting in an increased speed of muscle contraction [53]. O3FA lipid changes of neural membranes may affect endocytosis, exocytosis, membrane fusion, and neurotransmitter uptake and release [54]. A recent study in rodents has demonstrated that O3FA supplementation resulted in an increase in peripheral nerve function after injury [55]. Therefore, neuronal function declines with age and O3FA supplementation may provide a strategy to ameliorate some of this decline, although more research into this area is needed. Potential reasons for why we did not observe any changes over the supplementation period in grip strength may be because the majority of the participants already had healthy values. Also, it may be that a physical stimulus is required to illicit these effects and O3FAs may only be of benefit for increasing strength when given in addition to physical activity. Further, it may be more advantageous to measure lower body strength (quadriceps) than handgrip strength, since strength declines with age occur to a greater extent in the muscles of the lower body [56].

### Influence of Supplementation on Blood and Cardiovascular Measures

Plasma TGs were the only blood measure that changed significantly with FO supplementation, where the FO group had a 29% decrease (0.29 ± 0.08 mmol/L), while no significant differences occurred in the PL group. The effects of fish oil supplementation on TGs are well documented, and our results are similar to changes reported by numerous other studies, with an average decrease of approximately 30% [57]. Other metabolic effects of fish oils on lipid profile have been reported and include a small increase of LDL and HDL cholesterol [11]. Although the FO group followed these blood trends, no significant changes were apparent. Similarly, no significant changes in insulin and glucose levels were found. This finding is consistent with the majority of data reporting that omega-3 fatty acids do not have a role on glucose homeostasis [11].

Improvements in cardiovascular measures were evident with FO supplementation. The significant changes in RHR were noticeable after 6 wk of FO supplementation, where the females experienced a 5% (3 bpm) decrease. Similar results were found during exercise where a decrease of 2% (2 bpm) occurred for the females. The influence of FO on HR variability is well documented, with the greatest decreases occurring in older adults and in those with high resting HR [56]. Our cohort had decreases in resting HR that were similar to other studies in healthy older adults [57, 58]. For BP, no significant difference from baseline measures was found. However, meta-analyses of randomized, controlled trials have reported that intakes in the range of 2–3 g/d of FO resulted in a decrease in both SBP and DBP, especially in adults older than 45 yr [13, 59].

### Limitations and Future Directions

The majority of O3FA research in older adults is often investigated on diseased populations, and little is known about the effects of supplementation on physical and metabolic markers in healthy older individuals. The physical measures (body composition, cardiovascular, and blood) of our cohort appear to be similar to Canadian population data of adults matched for age and sex [60–63]. Therefore, the research in this paper appears to be applicable for the ‘average’ Canadian older female.

Much remains unknown regarding the potential benefits of O3FAs, especially in community-dwelling older adults. Future research should also aim to test a greater number of participants and include a longer period of supplementation (ie. 1 yr) to determine whether the increase in metabolic rate results in changes in more robust changes in body composition. In addition, the consumption of 5 g/d of total FO is difficult to maintain for many older adults, due to increased digestive issues (gastrointestinal discomfort) and the size of the capsules. Further, we did not determine whether all of the older females in the study were post-menopausal. Determining the optimal dose of FO required to illicit the metabolic and physical benefits is needed.

## Conclusion

We have demonstrated that FO supplementation (2 g/d EPA, 1 g/d DHA) for 12 wk in healthy community dwelling older females increased metabolic rate and fat oxidation at rest and during exercise, decreased resting and exercise HR and increased lean mass and physical function. The mechanisms behind the increases in resting and low exercise intensity metabolic rates await invasive measurements in future studies.

## Supporting Information

S1 CONSORT Checklist CONSORT Checklist. (DOCX)

S1 File REB Approval Letter. (DOCX)

S1 Protocol Trial Protocol. Application to Involve Humans in Research.
(DOC)
