# Supplementary material for: Omega-3 Fatty Acid Supplementation for 12 Weeks Increases Resting and Exercise Metabolic Rate in Healthy Community-Dwelling Older Females
Source: PLoS One. 2015 Dec 17;10(12):e0144828. doi: 10.1371/journal.pone.0144828 (PMC4682991; doi:10.1371/journal.pone.0144828)
Supplement: S1 CONSORT Checklist — (DOCX) [file pone.0144828.s001.docx]

**Consort Checklist**

**Title and Abstract**

**1a. Title**

Omega-3 Fatty Acid Supplementation for 12 Weeks Increases Resting and Exercise Metabolic Rate in Healthy Community-Dwelling Older Females: A Randomized Controlled Trial

**1b. Abstract**

Critical among the changes that occur with aging are decreases in muscle mass and metabolic rate and an increase in fat mass. These changes may predispose older adults to chronic disease and functional impairment; ultimately resulting in a decrease in the quality of life. Research has suggested that long chain omega-3 fatty acids, found predominantly in fatty fish, may assist in reducing these changes. The objective of this study was to evaluate the effect of fish oil (FO) supplementation in a cohort of healthy, community-dwelling older females on 1) metabolic rate and substrate oxidation at rest and during exercise; 2) resting blood pressure and resting and exercise heart rates; 3) body composition; 4) strength and physical function, and; 5) blood measures of insulin, glucose, c-reactive protein, and triglycerides. Twenty-four females (66 ± 1 yr) were recruited and randomly assigned to receive either 3g/d of EPA and DHA or a placebo (PL, olive oil) for 12 wk. Exercise measurements were taken before and after 12 wk of supplementation and resting metabolic measures were made before and at 6 and 12 wk of supplementation. The results demonstrated that FO supplementation significantly increased resting metabolic rate by 14%, energy expenditure during exercise by 10%, and the rate of fat oxidation during rest by 19% and during exercise by 27%. In addition, FO consumption lowered triglyceride levels by 29% and increased lean mass by 4% and functional capacity by 7%, while no changes occurred in the PL group. In conclusion, FO may be a strategy to improve age-related physical and metabolic changes in healthy older females.

**Introduction**

**2a. Background**

The proportion of seniors in Canada is predicted to increase from 13% of the total population in 2005 to 25% in 2036 [1]. With age, adults experience metabolic and physical changes, including increases in heart rate (HR), blood pressure, and fat mass (FM), and decreases in resting metabolic rate (RMR), lean body mass (LM), and physical function [2, 3]. These changes predispose older adults to age-related diseases and functional impairment, ultimately resulting in an overall decrease in the quality of life (QOL). There are several strategies to maintain the health and independence of older adults, including increasing cognitive and physical activity, exercise, and optimizing nutrition [4, 5].

A family of nutrients of interest are the long-chain omega-3 fatty acids (O3FAs); specifically, eicosapentaenoic acid (EPA, C20:5n-3) and docosahexaenoic acid (DHA, C22:6n-3). Since the body can only synthesize limited amounts of EPA and DHA from alpha-linolenic acid (C18: 3n-3), these fatty acids must be obtained from the diet or through supplementation [6]. The main dietary source of EPA and DHA is seafood, with the highest concentrations found in fatty fish. The American Heart Association (AHA) and Health Canada (HC) recommend that adults consume 500 mg/d of EPA and DHA (~2 servings/wk or ~8 oz of fish/wk) [7, 8]. However, the mean intake in Western society is ~135 mg/d (~2 servings of fish/mo) [9]. Research in our laboratory also observed low intakes of O3FAs in affluent populations (~230 mg/d), despite selecting healthier foods [10].

The benefits of O3FAs are far reaching due to their integration into cell membranes. The current dietary recommendations have been developed on the premise of reducing the risk factors associated with cardiovascular disease [8, 11], with the positive health benefits often seen with doses higher (3-4 g/d) than the AHA and HC recommendations. Decreases in resting blood pressure and HR [12, 13] and improvements in the blood lipid profile (triglyceride, total cholesterol, LDL-cholesterol) [11] have been extensively researched in populations with disease or at high risk of disease.

Research has documented a decrease in RMR, and a shift in body composition towards decreased LM and increased adiposity with aging [2, 14]. Low muscle mass and a high FM are associated not only with an increased risk of many age-related disease processes, but also with mobility impairment. The decrease in RMR and LM begins around the 3rd decade of life and result in declines of ~1-2%/decade for RMR [15] and ~0.26 - 0.56%/annum for LM [14]. The decline in RMR and LM are likely due to numerous factors, which include declining physical activity and nutrient intake, such as insufficient protein intake [16]. Skeletal muscle is responsible for ~20% of the metabolic rate at rest and up to ~80% of the energy consumption during exercise [17]. Research has suggested that O3FA intake, particularly EPA and DHA may increase RMR during rest and exercise in healthy adults, and substrate oxidation to favour a greater usage of fat [18, 19]. We recently demonstrated an increase in RMR after 12 wk of EPA and DHA supplementation and the incorporation of these fatty acids into the sarcolemmal and mitochondria membranes of human skeletal muscle of young healthy males [20, 21].

The incorporation of EPA and DHA into cell membranes may impact energy metabolism in many ways, including the regulation of cellular processes by altering gene expression, by acting as a ligand for peroxisome proliferator-activated receptors (PPARs) [22]. PPARs play an important role in energy homeostasis by regulating a wide array of genes involved in lipid metabolism [23].

**2b. Objectives**

We hypothesized that FO supplementation would result in: 1) an increased metabolic rate and a greater reliance on fat oxidation both at rest and during exercise; 2) a decrease in resting blood pressure and resting and exercise HR; 3) a decrease in adiposity and an increase in LM; 4) an increase in handgrip strength and physical function, and; 5) more healthy blood measures of high sensitivity c-reactive protein (hs-CRP) and triglycerides (TGs). A placebo (PL) group supplemented with 3 g/d of olive oil was included to control for the effects of confounding variables.

**Methods**

**3a. Trial Design**

Randomized, single blinded clinical trial. Following the third visit, the participants were matched by age, (BMI), and medication use, and were randomly assigned in a single-blinded manner to one of two supplement groups: fish oil (FO, 12 females) or placebo (PL, 12 females). We recruited as many participants as were willing to participate over a 6 month period.

**3b. Changes to trial design**

There were no changes to the methods after trial commencement.

**4a. Participants**

Females who met the following inclusion criteria were included in the study: (1) between the ages of 60-76 yr; (2) good cognitive status, as determined by a score >25/30 on the Mini Mental State Exam [25]; (3) consumed one meal or less of fish/wk and did not take a omega-3 supplement; (4) took no prescription medications or very low dose medications (hypertension, hypercholesterolemia, hormonal); and (5) absence of any self-reported medical diagnoses that entailed functional impairment. Following Research Ethics Board approval from the University of Guelph, both oral and written informed consent was obtained from all participants. Consent was also attained from the participant’s medical practitioner. For the study duration, participants were instructed to maintain their current diet and physical exercise regime. Twenty four females completed the study, as two of the females dropped out prior to supplementing, due to difficulty with the time commitment and personal issues with the metabolic and blood measures.

**4b. Study settings**

All data was collected at the University of Guelph (Guelph, ON)

**5. Interventions**

Experimental Protocol

After screening and recruitment, the participants reported to the laboratory on 7 separate occasions over a 14 wk period (Figure 1). Prior to all visits, participants were instructed to abstain from athletic activities and consume a mutually agreed on ‘normal’ diet [~50% energy (E) carbohydrate (CHO), ~30% E fat, and ~20% E protein] on the preceding day. During the first visit, participants completed the Physical Activity Scale for the Elderly (PASE) questionnaire, anthropometric measures (height (Ht), body mass (BM), waist circumference (WC), body composition), cardiovascular (resting HR (RHR) and blood pressure) and blood measures (fasting insulin, glucose, hs-CRP, cholesterol, TG), handgrip strength, and physical capacity measures (Berg Balance, Dynamic Index, Timed Get Up and Go (TUG), and 30-Second Chair Stand (30-SCS)). Participants also completed a cycling practice trial on an electronically braked cycle ergometer (LODE Excalibur; Quinton Instrument, Groningen, The Netherlands) to determine the power output needed to maintain the participant’s HR within a zone of low intensity (40% of HR reserve, HRR). The HRR was calculated as HRR = (maximal HR– resting HR) + resting HR [26]. The participants also evaluated their cycling intensity during all exercise trials using the Rating of Perceived Exertion (RPE) Scale [27].

During the second visit, participants reported to the laboratory following a 12-hr overnight fast and were instructed to lay supine in a darkened room for 30 min. Participants provided breath samples during the last 15 min to measure RMR. The volume of oxygen consumed (VO2; mL/min) and carbon dioxide produced (VCO2; mL/min) were determined using a metabolic cart (MOXUS metabolic system; AEI Technologies, Pittsburgh, PA, US). HR was recorded every 5 min with a heart rate monitor (Polar Electro, Inc., Port Washington, NY, US). After the RMR measurement, body composition was analyzed for FM and fat-free mass or LM using bioelectrical impedance analysis (Bodystat 1500, FL, US), and a resting venous blood sample was taken.

At least 2 days later, participants reported to the laboratory for a third visit to complete a 30 min exercise trial. Participants were instructed to eat a mutually agreed upon breakfast (~50% E CHO, ~30% E fat, and ~20% E protein (~350 kcal)) 2 hr before arriving to the laboratory and drink 500 mL of water within the 2 hr before arrival to ensure hydration. The participant was asked to provide a detailed log of their breakfast and instructed to consume the same breakfast on the post supplementation testing day. The participants then completed 30 min of low intensity cycling exercise at the power output established from the first visit. HR was recorded every 5 min and 4 min respiratory gas measurements were collected at the end of every 10 min (6-10, 16-20, 26-30 min). Following the third visit, the participants were matched by age, (BMI), and medication use, and were randomly assigned in a single-blinded manner to one of two supplement groups: fish oil (FO, 12 females) or placebo (PL, 12 females). The FO group took 5 g/d of FO (Omega-3 Complete, Jamieson Laboratories Ltd., Windsor, ON, CA) administered in 5 capsules, with each capsule providing 400 mg of EPA and 200 mg of DHA (daily total, 2 g EPA and 1 g DHA). The PL group took 3 g/d of olive oil (Swanson EFAs, Certified Organic Olive Oil, Swanson Health Products, Fargo, ND, US) administered in 3 capsules. To reduce any minor side-effects of the oils (burping, indigestion), the participants were instructed to take the supplements frozen and with meals; with the FO group taking 1 capsule at breakfast, 2 at lunch, and 2 at supper; and the PL group taking 1 capsule at each meal. To encourage compliance, the first month of supplements were provided in daily packets. After 4 wk the capsules were allotted in weekly amounts. In addition to picking up the supplements, participant compliance was encouraged with periodic phone calls and email reminders. Following 6 wk of supplementation, participants completed the 12 hr overnight fasted and resting protocol from the second lab visit, without the blood sample. After 12 wk of supplementation, the participants repeated the protocol from visits 2, 3, and 4, with the exception of the balance measures from visit 2. Dietary and physical activity compliance were assessed by monitoring records at the start and end of the study. Dietary intake was assessed by the completion of a three day food record and physical activity by the PASE questionnaire pre and post supplementation [28].

At the end of the study the participants were asked which supplement group they believed they were in. The majority of the participants taking FO (65%) and PL (55%) correctly identified their supplement group. The only supplementation symptoms reported were belching and heartburn, as 70% of the FO participants reported belching on ~1-2 d/wk and 10% reported heartburn on ~3-4 d/wk. Overall, the PL supplement was well tolerated, with only one participant reporting heartburn on ~3-4 occasions/wk during the last 6 wk of supplementation.

Physical Measures

All body composition (Ht, BM, WC) and grip strength measures were conducted as outlined in the Canadian Physical Activity, Fitness and Lifestyle Approach (CPAFLA) [29]. Briefly, Ht was measured to the nearest 0.1 cm using a vertical metric wall tape and a horizontal flat edge, BM was measured to the nearest 0.1 kg on a calibrated digital scale (Health O Meter; Bridgeview, IL, US) and WC was measured to the nearest 0.5 cm, and was taken at the top of the iliac crests using an anthropometric tape. BMI was calculated as BM/Ht2.

Bioelectrical Impedance Analysis (Bodystat 1500, FL, US) was completed directly after the 12-hr fasted and RMR measures, where the participant continued to lay supine with limbs abducted, and leads were attached according to manufacturer’s instructions. Fat free mass index (FFMI) was calculated using LM and standardizing for height (LM (kg)/ Ht2 (m2)) [30]. Isometric handgrip strength was measured using a hydraulic hand-held dynamometer (Vernier Jamar; Sammons Preston Rolyan; Nottinghamshire, ENG, UK). Three measurements per hand were taken and the participant alternated hands between measurements to allow ~30 s of rest. The highest measurement for each hand was added to achieve the combined grip strength (CGS) value.

To assess functional capacity, the Berg Balance, Dynamic Gait Index, TUG, and the 30- 30-SCS tests were employed as described elsewhere [30-32]. Since the functional capacity, as determined by the Berg Balance and Dynamic Index tests, of the cohort was high, we only repeated the TUG test and the 30-SCS post-supplementation.

Cardiovascular and Blood Measures

Resting systolic and diastolic blood pressures (SBP, DBP; mmHg) were measured using a blood pressure monitor (OMRON IntelliSense; Model HEM-907XL; OMRON Healthcare, IL, US). Participants were seated with their left arm resting on a table for 3 min prior to three blood pressure measurements taken 1 min apart. There was no significant difference between the three resting values so the mean of all measurements were used for data analysis.

After a 12 hr overnight fast, venous blood was collected and analyzed for serum glucose, insulin, hs-CRP, fatty acids, and TGs (mmol/L). The bloods were analyzed at LifeLabs Medical Laboratory Services (Guelph, ON, CA)

Metabolic Calculations

For both the rest and exercise trials, the VO2 and VCO2 were measured and used to calculate the respiratory exchange ratio (VCO2/VO2, RER) and whole body carbohydrate oxidation (CHO Ox) and fat oxidation (Fat Ox) by using the non-protein RER table and the following equations:

CHO Ox (g) = (4.585 x VCO2) – (3.226 x VO2); and

Fat Ox (g) = (1.695 x VO2) – (1.701 x VCO2).

The RMR or energy expenditure (Energy Ex) was calculated using the thermal equivalent of VO2 consumed based on non-protein respiratory quotient table and the following equation [33]:

Energy Ex (Kcal) = VO2 (L/min) x RER cal equiv (Kcal/L) x Time (min).

Questionnaires

The participants completed the PASE questionnaire, designed to measure the amount of physical activity engaged in over the past 7 d, with higher scores indicative of greater amounts of daily activity [28].

Assessment of Dietary Intake

The participants were asked to record their food and beverage consumption using a multiple-day food record (version 3; Fred Hutchison, WA, US) on 3 consecutive days, which included 2 weekdays and 1 weekend day. Detailed instructions were provided to the participants to ensure accurate dietary intake recording. The dietary information was entered into the Food Processor SQL-ESHA database version 10.8.0 (ESHA Research, Salem, OR, US).

Statistical Analysis

After determining data normality and variance homogeneity, an analysis of variance ANOVA (2-way repeated measures) and Tukey’s post hoc tests were used to evaluate the effect of time on the physical and metabolic measures. Data are presented as means ± SEMs. Statistical significance was accepted as p<0.05 for all tests. All statistics were computed using PASW Statistics 19.0.1 for Windows (Chicago, IL, US).

**6a. Outcomes**

This study demonstrated that FO supplementation (2 g EPA, 1 g DHA/d) for 12 wk in community-dwelling older female adults resulted in: 1) increased metabolic rate and fat oxidation both at rest and during exercise; 2) decreased resting and exercise HR; 3) increased LM and physical function, and; 4) decreased fasted blood TGs. In addition, supplementation with a PL (3 g olive oil/d) had no effect on any measures.

**6b. Changes to outcomes**

No changes to trial outcomes were made after the trial commenced.

**7a. Sample size**

We recruited as many participants as were willing to participate over a 6 month period.

**7b. Interim analyses and stopping guidelines**

Participants were instructed to report any side effects of supplementation to the researchers and were well-informed that they were able to decline participation in the study at any point.

**8a. Randomisation: sequence generation**

N/A

**8b. Randomisation: type**

Following the third visit, the participants were matched by age, (BMI), and medication use, and were randomly assigned in a single-blinded manner to one of two supplement groups: fish oil (FO, 12 females) or placebo (PL, 12 females).

**9. Randomisation: allocation concealment mechanism**

**10. Randomisation: implementation**

SLL and LSS generated the allocation sequence, SLL enrolled participants, and SLL and LLS assigned participants to interventions.

**11a. Blinding**

Participants were blinded to their supplementation group (fish oil or placebo). Following the third visit, the participants were matched by age, (BMI), and medication use, and were randomly assigned in a single-blinded manner to one of two supplement groups: fish oil (FO, 12 females) or placebo (PL, 12 females). To encourage compliance, the first month of supplements were provided in daily packets. After 4 wk the capsules were allotted in weekly amounts. In addition to picking up the supplements, participant compliance was encouraged with periodic phone calls and email reminders.

**11b. Similarity of interventions**

N/A

**12a. Statistical methods**

After determining data normality and variance homogeneity, an analysis of variance ANOVA (2-way repeated measures) and Tukey’s post hoc tests were used to evaluate the effect of time on the physical and metabolic measures. Data are presented as means ± SEMs. Statistical significance was accepted as *p*<0.05 for all tests. All statistics were computed using PASW Statistics 19.0.1 for Windows (Chicago, IL, US).

**12b. Additional analyses**

N/A

**Results**

**13a. Participant Flow**

12 participants were assigned to the fish oil (FO) group and 12 participants were assigned to the placebo (PL) group. All participants were analyzed for the health and physical measures and the metabolic measures. Only 6 FO participants and 11 PL participants participated in the blood draw measures.

**13b. Losses and exclusions**

There were no loses or exclusions after randomization. However, 6 FO participants and 1 PL participants declined participation in the blood draw measures due to being uncomfortable with blood drawn.

**14a. Recruitment**

January 2013 to October 2013.

**14b. Reason for stopped trial**

The researchers allocated a 10 month period for recruitment and testing of participants.

**15. Baseline Data**

**Table 1.** Participant health and physical measures at 0 and 12 wk of supplementation with placebo or fish oil.

|  | Placebo (n=12) | | Fish Oil (n=12) | |
| --- | --- | --- | --- | --- |
|  | **0 Wk** | **12 Wk** | **0 Wk** | **12 Wk** |
| Body Composition & Cardiovascular |  |  |  |  |
| Body Mass (kg) | 69.1 ± 3.0 | 69.0 ± 3.1 | 72.9 ± 3.0 | 73.3 ± 3.7 |
| Body Mass Index (kg/m^2^) | 26.3 ± 1.0 | 26.3 ± 1.1 | 27.9 ± 1.3 | 28.0 ± 1.2 |
| Waist Circumference (cm) | 91.4 ± 3.1 | 90.2 ± 2.7 | 92.6 ± 2.3 | 92.1 ± 2.9 |
| Fat Mass (kg) | 29.6 ± 1.9 | 28.9 ± 2.0 | 32.6 ± 2.0 | 31.3 ± 2.2 y, July 2.ipants, 41 (95.3%) completed the study. The 2 participants that did not complete the study dropped out due to u |
| Lean Mass (kg) | 39.5 ± 1.4 | 40.1 ± 1.6 | 40.3 ± 1.2 | 41.9 ± 1.3* |
| Systolic Blood Pressure (mmHg) | 119 ± 3.3 | 116 ± 4.9 | 117 ± 4.8 | 115 ± 3.8 |
| Diastolic Blood Pressure (mmHg) | 72 ± 1.9 | 72 ± 2.6 | 70 ± 3.5 | 66 ± 2.6 |
| Function & Strength |  |  |  |  |
| Combined Grip Strength (kg) | 57.4 ± 2.3 | 57.1 ± 2.4 | 49.9 ± 2.8 | 51.5 ± 3.4 |
| Timed Up and Go Test (s) | 7.3 ± 0.2 | 7.1 ± 0.2 | 7.6 ± 0.2 | 7.1 ± 0.2* |
| 30-Second Sit To Stand (# Completed) | 15 ± 0.9 | 17 ± 1.1 | 15 ± 1.7 | 17 ± 1.9 |
| PASE | 120 ± 21 | 124 ± 22 | 149 ±15 | 152 ± 18 |
| Dietary Intake |  |  |  |  |
| Total Energy Intake (kcal) | 1926 ± 166 | 2009 ± 155 | 1867 ± 107 | 1924 ± 140 |
| Fat (g) | 64 ± 6 | 71 ± 9 | 59 ± 6 | 64 ± 6 |
| Protein (g) | 72 ± 5 | 81 ± 6 | 81 ± 5 | 86 ± 4 |
| Carbohydrate (g) | 258 ± 28 | 261 ± 17 | 253 ± 25 | 269 ± 21 |
| Fasted Blood | **n=6** |  | **n=11** |  |
| Insulin (pmol/L) | 65.6 ± 21.3 | 61.2 ± 15.8 | 52.1 ± 4.4 | 49.4 ± 6.6 |
| Glucose (mmol/L) | 5.0 ± 0.3 | 4.96 ± 0.26 | 5.04 ± 0.13 | 5.12 ± 0.13 |
| C-Reactive Protein (mg/L) | 1.75 ± 0.33 ^+^ | 1.67 ± 0.25 ^++^ | 3.28 ± 0.70 | 3.29 ± 0.59 |
| Triglycerides (mmol/L) | 1.19 ± 0.15 | 1.13 ± 0.13 | 1.30 ± 0.14 | 1.01 ± 0.14 * |
|  | **n=6** |  | **n=9** |  |
| EPA (% of total fatty acids) | 0.85 ± 0.13 | 1.04 ± 0.13 | 0.86 ± 0.10 | 5.97 ± 0.62 * |
| DHA (% of total fatty acids) | 1.73 ± 0.22 | 1.92 ± 0.27 | 1.77 ± 0.09 | 3.79 ± 0.19 * |
| EPA (absolute %) | 14.50 ± 2.40 | 19.71 ± 2.49 | 13.85 ± 1.82 | 88.17 ± 9.34 * |
| DHA (absolute %) | 28.99 ± 3.89 | 36.48 ± 5.52 | 27.73 ± 1.66 | 57.47 ± 3.96 * |

Data are means (±SE). PASE=physical activity score for the elderly questionnaire; EPA=eicosapentaenoic acid; DHA = dicosahexaenoic acid. Significant difference *within groups at 0 and 12 wk, and between groups at ^+^0 wk and ^++^12 wk.

**16. Numbers analysed**

For the fish oil (FO) and the placebo (PL) group, 12 participants in each group were analyzed for the health, physical, and metabolic measures. For the bloods, however, only 6 of the 12 participants in the FO group were analyzed and 11 of the 12 in the PL group were analysed.

**17a. Outcomes and estimation**

*Participant Characteristics*

The participant data indicated that the cohort was generally in good health, although the body composition data denoted that the cohort was overweight (Table 1). The mean FFMI values indicated that the participants had healthy amounts of skeletal muscle. The cohort was also high functioning according to the balance, TUG, and 30-SCS tests, and possessed healthy CGS values (Table 1). The physical activity level of the cohort (PASE score) was above average in comparison to sex and age matched normative data for older adults [28]. The nutrition data demonstrated that the total energy intake for the females was indicative of a low to moderate level of daily activity, and low levels of daily activity [34]. The participants also consumed healthy amounts of fat, CHO and protein [35], and the average energy intake did not significantly change over the supplementation period. Compliance was demonstrated by the significant increase in the percent of EPA (0 wk, 0.91 ± 0.10; 12 wk, 6.0 ± 0.62) and DHA (0 wk, 1.8 ± 0.09; 12 wk, 3.8 ± 0.19) in the FO group, while serum levels of EPA (0 wk, 0.85 ± 0.13; 12 wk, 1.04 ± 0.13) and DHA (0 wk, 1.73 ± 0.22; 12 wk, 1.92 ± 0.27) in the PL group did not change. The cardiovascular data indicated that the mean SBP and DBP were in the healthy range (Table 2). Further, the fasted blood data indicated that all of the participants had healthy insulin, glucose, and TG values. The hs-CRP and TC values indicated that a low level of risk of cardiovascular disease was evident (Table 1). Finally, the use of medication was low in the cohort, with 42% taking any form of low-dose medications.

The groups were well matched for the baseline physical and metabolic measures (p > 0.05), with the exception of hs-CRP values, where the FO group had significantly higher baseline values of hs-CRP than the PL group (Table 2).

*Influence of Supplementation on Physical and Blood Measures*

After 12 wk of supplementation, a significant increase in LM of 1.6 ± 0.7 kg and decrease in TUG speed of 0.5 ± 0.2 s was found in the FO group, while no significant changes were found in the PL group for LM or TUG. TG values also significantly decreased by 0.29 ± 0.07 mmol/L for the FO females, while no significant change was found in the PL group. Finally, there were no significant changes in any of the other body composition, physical function and strength measures (Table 1), or in the other cardiovascular and blood values for both the FO and PL groups over time (Table 2).

*Influence of Supplementation on Resting Metabolic Measures*

Significant increases in VO_2_ of 20.1 ± 8.9 mL/min after 6 wk, and 27.6 ± 8.0 mL/min after 12 wk of supplementation occurred in response to FO intake (Table 2, Figure 2), while no significant changes occurred in the PL group. Resting VCO_2_ followed a similar trend, with a significant increase of 18.4 ± 5.73 mL/min for the FO group after 12 wk of supplementation while no changes were found in the PL group (Table 2). The changes in VO_2_ resulted in a significant increase in RMR of 0.10 ± 0.04 kcal/min at 6 wk and 0.13 ± 0.04 kcal/min after 12 wk of FO supplementation (Table 2, Figure 4). No changes in RMR were evident in the PL group. Finally, RMR remained significantly increased in the FO group when normalized for BM and LM (Table 2, Figure 4).

The substrate oxidation data indicated that FO supplementation resulted in a significant increase in the rate of Fat Ox of 15.5 ± 5.7 mg/min, while no significant changes were found in the PL group after 12 wk of supplementation (Table 2, Figure 3). Further, no significant changes were found in the rate of CHO Ox for the FO and PL groups. Finally, FO supplementation decreased RHR by 3 ± 1 bpm after 6 wk without further decreases at 12 wk. RHR remained significantly unchanged in the PL group (Table 2).

*Influence of Supplementation on Exercise Metabolic Measures*

The average power output during the exercise trial was 36 ± 4 W for the PL group and 35 ± 6 W for the FO group. VO_2_ significantly increased by 99.2 ± 19.7 mL/min for the FO group after 12 wk of supplementation, while no changes were evident in the PL group (Table 3, Figure 2). Exercise VCO_2_ followed a similar trend where a significant increases of 60.7 ± 20.1 mL/min occurred in response to FO supplementation at 12 weeks while no changes were found in the PL group (Table 3). RER was unchanged in the FO and PL groups. Total Energy Ex increased significantly by 13.8 ± 2.5 kcal in the FO group only (Table 3, Figure 4). When expressed as a rate, an increase of 0.46 ± 0.08 kcal/min for the FO group occurred, with no significant changes PL group. Finally, Energy Ex remained significantly increased when normalized for BM and LM (Table 3).

FO supplementation significantly increased total Fat Ox by 1.9 ± 0.6 g (Table 3). When calculated as a rate, an increase of 0.07 ± 0.02 g/min for the FO group occurred (Figure 3), while no significant changes were found in the PL group (Table 3). Further, no significant changes were found in total CHO Ox or in the rate of CHO Ox over 12 wk of FO and PL supplementation.

Finally, exercise HR was decreased by 3 ± 1 bpm in response to FO supplementation (Table 3), with no significant changes in the PL group.

**17b. Binary outcomes**

N/A

**18.  Ancillary analyses**

N/A

**19. Harms**

The only supplementation symptoms reported were belching and heartburn, as 70% of the FO participants reported belching on ~1-2 d/wk and 10% reported heartburn on ~3-4 d/wk. Overall, the PL supplement was well tolerated, with only one participant reporting heartburn on ~3-4 occasions/wk during the last 6 wk of supplementation.

**Discussion**

**20. Limitations**

The majority of O3FA research in older adults is often investigated on diseased populations, and little is known about the effects of supplementation on physical and metabolic markers in healthy older individuals. The physical measures (body composition, cardiovascular, and blood) of our cohort appear to be similar to Canadian population data of adults matched for age and sex [60-63]. Therefore, the research in this paper appears to be applicable for the ‘average’ Canadian older female.

Much remains unknown regarding the potential benefits of O3FAs, especially in community-dwelling older adults. Future research should also aim to test a greater number of participants and include a longer period of supplementation (ie. 1 yr) to determine whether the increase in metabolic rate results in changes in more robust changes in body composition. In addition, the consumption of 5 g/d of total FO is difficult to maintain for many older adults, due to increased digestive issues (gastrointestinal discomfort) and the size of the capsules. Determining the optimal dose of FO required to illicit the metabolic and physical benefits is needed.

**21. Generalisability**

The results demonstrated that FO supplementation significantly increased resting metabolic rate by 14%, energy expenditure during exercise by 10%, and the rate of fat oxidation during rest by 19% and during exercise by 27%. In addition, FO consumption lowered triglyceride levels by 29% and increased lean mass by 4% and functional capacity by 7%, while no changes occurred in the PL group. In conclusion, FO may be a strategy to improve age-related physical and metabolic changes in healthy older females.

**22. Interpretation**

We have demonstrated that FO supplementation (2 g/d EPA, 1 g/d DHA) for 12 wk in healthy community dwelling older females increased metabolic rate and fat oxidation at rest and during exercise, decreased resting and exercise HR and increased lean mass and physical function. The mechanisms behind the increases in resting and low exercise intensity metabolic rates await invasive measurements in future studies.

**Other information**

**23. Registration**

Clinical Trial (NCT01734538)

**24. Protocol**

In the paper or Supporting Documents

**25. Funding**

This funding was supported by a Natural Sciences and Engineering Research Council of Canada Discovery grant to LLS.
