# Supplementary material for: Omega-3 Fatty Acid Supplementation for 12 Weeks Increases Resting and Exercise Metabolic Rate in Healthy Community-Dwelling Older Females
Source: PLoS One. 2015 Dec 17;10(12):e0144828. doi: 10.1371/journal.pone.0144828 (PMC4682991; doi:10.1371/journal.pone.0144828)
Supplement: S1 File — (DOCX) [file pone.0144828.s002.docx]

| **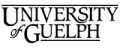** | **RESEARCH ETHICS BOARD**  Certification of Ethical Acceptability of Research  Involving Human Participants |
| --- | --- |

**APPROVAL PERIOD:** **December 4, 2012 to** **December 4, 2013**

**REB NUMBER:** **12OC014**

**TYPE OF REVIEW: Full Board**

**RESPONSIBLE FACULTY:** **LAWRENCE SPRIET**

**DEPARTMENT:** **Human Health & Nutritional Sciences**

**SPONSOR:** **NSERC DISCOVERY GRANT (RG)**

**JAMIESON LABORATORIES**

**TITLE OF PROJECT:**  **The effect of 12 weeks of omega-3 fatty acid**

**supplementation on metabolic and physical health**

**parameters in older adults.**

The members of the University of Guelph Research Ethics Board have examined the protocol which describes the participation of the human subjects in the above-named research project and considers the procedures, as described by the applicant, to conform to the University's ethical standards and the Tri-Council Policy Statement.

The REB requires that you adhere to the protocol as last reviewed and approved by the REB. The REB must approve any modifications before they can be implemented. If you wish to modify your research project, please complete the Change Request Form.If there is a change in your source of funding, or a previously unfunded project receives funding, you must report this as a change to the protocol.

Adverse or unexpected events must be reported to the REB as soon as possible with an indication of how these events affect, in the view of the Responsible Faculty, the safety of the participants, and the continuation of the protocol.

If research participants are in the care of a health facility, at a school, or other institution or community organization, it is the responsibility of the Principal Investigator to ensure that the ethical guidelines and approvals of those facilities or institutions are obtained and filed with the REB prior to the initiation of any research protocols.

The Tri-council Policy Statement requires that ongoing research be monitored by, at a minimum, a final report and, if the approval period is longer than one year, annual reports. Continued approval is contingent on timely submission of reports.

**Membership of the Research Ethics Board:** B. Beresford, *Ext.*; F. Caldwell, *Physician;* C. Carstairs, *COA;* S. Chuang, *FRAN (alt);* K. Cooley*, Alt. Health Care;* J. Clark, *PoliSci (alt);* J. Devlin, *OAC;* J. Dwyer, *FRAN*; M. Dwyer, *Legal;* D. Dyck, *CBS*; D. Emslie, *Physician (alt);* B. Ferguson*, CME (alt);* H. Gilmour, *Legal (alt);* J. Goertz, *CME;* B. Gottlieb, *Psychology;* B. Giguere, *Psychology (alt);* S. Henson, *OAC (alt);* G. Holloway, *CBS;* L. Kuczynski, *Chair;* S. McEwen, *OVC (alt);*J. Minogue*, EHS;* A. Papadopoulos, *OVC;* B. Power*, Ext.;* V. Shalla, *SOAN (alt);* J. Srbely, *CBS (alt)*; R. Stansfield, *SOAN*; K. Wendling, *Ethics.*

Approved: Date: _______Dec 4, 2012__________

per

Chair, Research Ethics Board
