# Supplementary material for: Omega-3 Fatty Acid Supplementation for 12 Weeks Increases Resting and Exercise Metabolic Rate in Healthy Community-Dwelling Older Females
Source: PLoS One. 2015 Dec 17;10(12):e0144828. doi: 10.1371/journal.pone.0144828 (PMC4682991; doi:10.1371/journal.pone.0144828)
Supplement: S1 Protocol — Application to Involve Humans in Research. (DOC) [file pone.0144828.s003.doc]

**University of Guelph Research Ethics Board (REB)**

FACULTY AND GRADUATE

Application to Involve Human Participants in Research

**Please refer to the University of Guelph Research Ethics Guidelines, found at** [**http://www.uoguelph.ca/research/forms_policies_procedures/human_participants.shtml**](http://www.uoguelph.ca/research/forms_policies_procedures/human_participants.shtml) **before completing and submitting this application. If you have questions about this form, please contact the Research Ethics Coordinator, Sandra Auld at ext. 56606, or** [**reb@uoguelph.ca**](mailto:reb@uoguelph.ca)**.**

Send this form and all accompanying material by email, as attachments, to [**reb@uoguelph.ca**](mailto:reb@uoguelph.ca). One hard copy of the signed signature page should be forwarded to the Research Ethics Coordinator, Office of Research, University of Guelph, 437 University Centre, Guelph, ON, N1G 2W1.

If you want to change a previously approved protocol, please complete the “Change Request” form, available at <http://www.uoguelph.ca/research/forms_policies_procedures/human_participants.shtml> .

| **Date:** 2012-09-11 (yyyy-mm-dd) | **(For OR use only) Protocol#:** |
| --- | --- |

**SECTION A – GENERAL INFORMATION**

1. **Title of the Research Project:** The effect of 12 weeks of omega-3 fatty acid supplementation on metabolic and physical health parameters in older adults.

2. **Investigator Information**

.

|  | **Name & position** | **Dept./Address** | **Phone No.** | **E-Mail** |
| --- | --- | --- | --- | --- |
| **Faculty with Principal Responsibility*:** | Dr. Lawrence Spriet | HHNS | X 53745 | [lspriet@uoguelph.ca](mailto:lspriet@uoguelph.ca) |
| **Faculty:**  **Co-Investigator(s)** | Dr. David Mutch | HHNS | X 53322 | [dmutch@uoguelph.ca](mailto:dmutch@uoguelph.ca) |
| **Student: Investigator(s)** | Samantha Logan | HHNS | X 53907 | [slogan01@uoguelph.ca](mailto:slogan01@uoguelph.ca) |
| **Other:**  **Investigator(s)** | Chris Gerling  Jamie Whitfield  Kyle Boorsma  Tanya Holloway  Michael Zulyniak  Maude Perreault | HHNS | X 53907  X 58015 | [cgerling@uoguelph.ca](mailto:cgerling@uoguelph.ca)  [jwhitfie@uguelph.ca](mailto:jwhitfie@uguelph.ca)  [rboorsma@uoguelph.ca](mailto:rboorsma@uoguelph.ca)  [thollowa@uoguelph.ca](mailto:thollowa@uoguelph.ca)  [mzulynia@uoguelph.ca](mailto:mzulynia@uoguelph.ca)  [mperreau@uoguelph.ca](mailto:mperreau@uoguelph.ca) |

* **must be advisor of any student investigators**.

3.**Proposed Date** a) of commencement: November 15, 2012 b) of completion: November 15, 2013

Note: The commencement date should be the date the researcher expects to actually begin interacting with human participants (including recruitment). The completion date should be the date that the researcher expects that interaction with human participants, including any feedback or follow-up, will be complete.

4. **Indicate the location**(s) where the research will be conducted:

University of Guelph

Other (please specify site):

5. **Other Research Ethics Board Approval**

**Yes** **No**

1. Is this a multi-centred study?  X
2. Has any other institutional Ethics Board approved this project?  X
3. If **Yes**, please provide the following information:

Title of the project approved elsewhere:

Name of the Other Institution:

Name of the Other Board:

Date of the Decision:

A contact name and phone number for the other Board:

OR

A copy of the clearance certificate / approval

**Yes** **No**

1. Will any other Research Ethics Board be asked for approval?  X

If **Yes**, please specify: McMaster University, Faculty of Health Sciences

6. **Level of the Project**

Faculty Research

PhD Thesis X

Masters Thesis

Honours Thesis

Class Project

Internship

Practicum

Other (please specify):

7. **Funding of the Project**

**Yes No**

1. Is this project currently funded? X

b) Period of Funding: From To

1. Agency or Sponsor (funded or applied for)

CIHR:

NSERC: NSERC Discovery Grant

SSHRC:

Other (please specify):

Note: **Please specify the complete title of the funding source**. For example, “NSERC Discovery Grant”.

***NOTE: If the funding source changes, or if a previously unfunded project receives funding, you must submit a Change Form to the Research Ethics Coordinator.***

8. **Conflict of Interest**

1. Will the researcher(s), members of the research team, and/or their partners or immediate family members:
   1. Receive any personal benefits (for example a financial benefit such as remuneration, intellectual property rights, rights of employment, consultancies, board membership, share ownership, stock options etc.) as a result of or connected to this study? **Yes**  **No** X
   2. If **Yes**, please describe the benefits below. (Do not include conference and travel expense coverage, possible academic promotion, or other benefits which are integral to the general conduct of research.)

| Not Applicable |
| --- |

1. Describe any restrictions regarding access to or disclosure of information (during or at the end of the study) that the sponsor has placed on the investigator(s).

| Not Applicable |
| --- |

1. Discuss the possibility of commercialization of the research findings.

| Not Applicable |
| --- |

**SECTION B – SUMMARY OF THE PROPOSED RESEARCH**

9. **Rationale**

Describe the purpose and background rationale for the proposed project, as well as the hypotheses(is)/research questions to be examined.

| The biological membranes that surround a cell and its organelles are important to the overall function of the cell. Fatty acids are the main structural components of a membrane, and the presence of certain fatty acids can alter a membrane’s characteristics, which subsequently alters its function. Two fatty acids that are of particular interest to researchers are eicosapentanoic acid (EPA) and docosahexanoic acid (DHA). These omega-3 fatty acids have unique unsaturated structures, and their incorporation into biological membranes appears to elicit potent physiological effects. The body is unable to intrinsically synthesize these important fatty acids, so they must be obtained from the diet or through supplementation.  EPA and DHA supplementation has been reported to provide numerous positive health benefits, including decreased blood pressure and an improved blood lipid profile. Recent research in our laboratory has demonstrated in young men that resting metabolic rate was increased following a 12 week period of omega fatty acid supplementation of 3 g/day. Fat utilization was also increased and carbohydrate use was decreased both at rest and during a 1 hr moderate exercise challenge. These findings have positive implications for people who are interested in losing weight, but it not known whether these effects occur in older adults. With age, body composition changes (increase in fat mass and decrease in lean mass) occur which may negatively affect general health, particularly normal physical functioning and quality of living of the older adult. The potential role of omega 3 fatty acids to increase fat oxidation and increase resting metabolic rate may be a therapy for decreasing fat mass in the older adult. Research has suggested that omega 3 fatty acids may elicit an additional therapeutic role by reducing joint pain.  Another aspect of aging is a loss in lean body mass, which is associated with an attenuation of skeletal muscle protein synthesis in response to nutritional stimuli (amino acids and insulin). The weakened anabolic response is considered to be partly due to defects in the anabolic signaling cascade in the muscle. Research in older adults has reported an increase in muscle protein signaling with EPA and DHA supplementation in response to a nutritional challenge. However it is unknown whether the increase in protein signaling results in an eventual increase in lean body mass. The physical functioning of older adults is not only influenced by body composition changes, but also age-related neuronal changes that affect the velocity of axonal conduction and influence the ability of the muscle to generate torque and the rate at which the torque is developed. Muscular strength and functional capacity increases have been reported with chronic resistance exercise in a cohort of older women supplemented with EPA and DHA. However, the effect of EPA and DHA intake on physical strength in older adults with previously low EPA and DHA consumption, independent of exercise, is currently unknown.  Therefore, the purpose of this study is to determine the effects of 12 weeks of omega-3 supplementation at 3 g/day on metabolic and physical health parameters in community-dwelling older adults. We hypothesize that EPA and DHA supplementation will result in 1) a decrease in resting heart rate and blood pressure; 2) a more healthy blood profile; 3) an increase in resting metabolic rate; 4) a greater reliance on fat oxidation for energy both at rest and during exercise; and 5) a decrease in fat mass; and 6) an increase in lean mass, strength and physical function. |
| --- |

10. **Methodology**

Describe sequentially, and in detail, all procedures in which the research participants will be involved (e.g., paper and pencil tasks, interviews, surveys, questionnaires, physical assessments, physiological tests, time requirements etc.)

***Note: Attach a copy of all questionnaire(s), interview guides or other test instruments. These should be on University of Guelph letterhead if they are intended for public dispersal.***

| Participants will report to the laboratory on 8 occasions. The duration of this study will be ~16 weeks.  **Visit 1(~1 hr):** **Participant Screening Visit.** During Visit 1 the researcher will explain the study protocol and requirements to the potential participants. If the participant chooses to participate, a signed consent form will be attained (see Consent to Participate in Research). The participant will then complete the Mini-Mental State Examination (MMSE) to determine that the participant has normal cognition and is able to provide signed consent to participate in this research. We will follow 3-G-010 protocol available at <http://www.uoguelph.ca/research/sites/default/files/3-G-010_MMSE.1.pdf> to administer this sensitive test. The Participant Screening form and the Omega-3 questionnaire (Appendix A), and the PAR-Q will also be completed to determine eligibility to participate. The participants will also take home the 3 Day Dietary Log to determine EPA and DHA consumption (Appendix B). The medical consent letter must also be signed by the participant’s physician (Appendix A). Eligibility to participate will be determined by the participant responses, as outlined on each questionnaire (see Appendix A for screening questionnaires and forms). Participants will be given a phone call by the researchers to inquire whether medical consent was attained, and whether DHA and EPA foods were recorded in the completed medical consent letter and 3-Day Dietary Log.  **Visit 2 (1 week later, ~ 2 hrs):** Participants will complete the Physical Activity Scale for the Elderly (PASE) questionnaire, the Short Form 36 (SF-36) health survey, and the Short Form McGill Pain questionnaire (see Appendix C for questionnaires). After this time participants will undergo a battery of physical measurements. Participants will then undergo a battery of physical measures/tests: Height, weight, strength (handgrip), balance tests (Berg Balance and Dynamic Balance), and a physical function test (Timed Up and Go) (see Appendix D for physical test forms).  At this time participants will also be required to complete a cycling practice trial at a low to moderate power output (50-100 W) to ensure that the participant can complete 30 min of exercise. Participants will provide respiratory gas samples at 10 min intervals. Heart rate will also be measured to ensure participants are within their range for this particular intensity of exercise. The participant will be instructed to stop exercising if they experience nausea, dizziness, or any sense of general un-wellness. The participant will be consistently monitored for any signs of these stresses.  **Visit 3 (1 week later, ~1 hr):** Participants will arrive to the laboratory following an overnight fast (~10-12 hours). Upon arrival, participants will be instructed to lay supine on a bed for ~30 min, providing breath samples during the last 15 minutes. Resting heart rate and blood pressure will be taken, and fat mass and lean mass will be determined by bioelectrical impedance analysis. Waist circumference will be taken with a measuring tape at the top of the iliac crests. 20 mL of blood will be taken by inserting a single venipuncture in a peripheral arm vein by an experienced technician (Premila Sathasivam, James Turgeon or Jamie-Lee Munroe) and plasma levels of glucose, insulin, triglycerides, lipoproteins, and omega 3 fatty acids will be measured. The amount of blood taken in this study is ~5% of the blood taken when donating at the Canadian Blood Services.  **Visit 4 (~2 days later, ~1 hr duration):** Participants will be instructed to eat breakfast [~50% of energy (E%) from CHO, ~30 E% from fat, and ~20 E% from protein] 2 hr before arriving to the laboratory. Participants will be required to complete 30 min of low to moderate cycle exercise (50-100 W) and breath samples at 10 minute intervals will be taken throughout exercise. Heart rate measurements will also be taken continuously during exercise. The participant will be instructed to stop exercising if they experience nausea, dizziness, or any sense of general un-wellness. The participant will be consistently monitored for any signs of these stresses.  Prior to all visits, participants will abstain from athletic activities and consume a mutually agreed on ‘normal’ diet [~50 E% from CHO, ~30 E% from fat, and ~20 E% from protein] during the preceding day, and consume a light breakfast with this same proportion of macronutrients as described above.  Immediately following Visit 4, all participants will be put on a fish oil or corn oil (control or placebo group) supplementation protocol for 12 weeks. The supplementation protocol will require the participants to consume 5 capsules of Omega-3 Complete (Jamieson Laboratories Ltd., Windsor, Ontario) or 5 placebo capsules daily with meals, providing ~3000 mg of EPA + DHA per day - please see attached product information (Appendix E). The placebo and treatment supplements will be supplied free of charge by Jamieson Laboratories. During this time period, participants will be asked not to make any lifestyle changes (diet, exercise, etc.). Participant compliance will be encouraged with periodic phone call reminders, visits to pick up capsules and verified by capsule count at the conclusion of the supplementation period.  **Visit 5 (~6 wks later, ~1 hr):** During Visit 5 participants will complete the Visit 3 protocol without the blood draw.  **Visit 6 (~5 weeks later, ~2 hr):** During Visit 6 the participants will complete the anthropometry, strength, balance and physical function protocol from Visit 2. The participants will also complete the Omega-3 questionnaire, the PASE questionnaire, and take home a 3 Day Dietary Log to complete, to ensure that diet and physical activity remained consistent throughout the trial period. Participants will also complete the SF-36, and the SF-McGill Pain questionnaire to research the relationship between EPA and DHA supplementation and health status and perceived physical pain.  **Visit 7 (1 week later, ~1 hr duration):** During Visit 7 the participants will submit the 3 Day Dietary Log from Visit 4 and again complete the Visit 3 protocol.  **Visit 8 (at least 2 days later, ~1 hr duration):** During Visit 8 the participants will complete the Visit 4 protocol.  Upon the completion and analysis of this study we will provide the participants with a copy of their results and the interpretation of the results (See Appendix E for Participant Results form). We will also reveal the participant’s supplement group to the participant. A copy of the manuscript will also be available to all participants.  **Data analysis:** Independent samples t-tests will be used to determine if there are differences between the two groups (placebo and fish oil) for baseline measures questionnaire scores and physical measures. The paired t-test will be used to test for variability within groups for questionnaire scores and physical measures at pre and post supplementation. The repeated measures ANOVA will be used to test for variability within groups at pre supplementation, 6 weeks of supplementation, and post supplementation for resting metabolic rate. The Independent Samples t-test will be used to analyze the relationship of the physical measures and questionnaire scores between groups for questionnaire scores and physical measures at pre and post supplementation. The Two-Way ANOVA will be used to determine if there is a significant difference between groups and sex for the questionnaire scores and physical measures at pre and post supplementation, and for resting metabolic rate at pre, 6 weeks, and post supplementation. All statistics were computed using PASW Statistics 19.0.1 for Windows (SPSS, Chicago, IL). Statistical significance will be accepted as p<0.05 for all tests.  **Procedure for accounting for missing, unused, and spurious data:** Missing data will be described, for example, by presenting the number and percentage of individuals in the missing category. All data collected on forms will be used, since only essential data items will be collected. No data will be considered spurious in the analysis since all data will be checked and cleaned before analysis.  **Procedures for reporting any deviation(s) from the original statistical plan (any deviation(s) from the original statistical plan should be described and justified in protocol and/or in the final report, as appropriate):** Deviations from the original statistical place are unlikely. However, should they occur, then approval for such changes will be sought from the REB and these changes will be noted in the final report and papers.  **The selection of subjects to be included in the analyses (e.g., all randomized subjects, all dosed subjects, all eligible subjects, evaluable subjects):** All eligible subjects will be included in the analysis.  **Specification of the efficacy parameters and methods and timing for assessing, recording, and analyzing of efficacy parameters:** It is not appropriate to look at efficacy parameters due to non-invasive nature of the measures being performed and the market availability of the supplement.  **Safety protocols:** All personnel involved with the exercise testing portion of this study are CPR certified and trained in the safe use of the metabolic carts and all related equipment. Safety protocol for emergencies during testing is posted in the testing lab, including the phone extension for campus police and contact information for all CPR-certified personnel involved in the study, as well as others in the building. Exercise testing protocol adheres to the guidelines outlined by the Canadian Society for Exercise Physiology and the American College of Sports Medicine.  **Researcher Attestation:** The researcher will adhere to TCPS2, the protocol as cleared by the REB, and the principles established by ICH-GCP E6 as warranted.  **The University of Guelph will permit trial-related monitoring, audits, REB review, and regulatory inspection(s), providing direct access to source data/documents as required.** |
| --- |

11. **Experience**

What is your experience with this kind of research?

| The principal investigator (Dr. Spriet) has extensive experience with all of the measures listed in this study and the inherent risks. The student investigator (Samantha Logan) and the other investigators, as listed in Section A, are familiar with the questionnaires and physical function measures. A similar protocol involving omega 3 supplementation and respiratory measures is underway in our lab. In addition, the student Samantha Logan has experience with a similar study evaluating questionnaire scores (Short-Form 36, Physical Activity Score for the Elderly, etc.) and testing physical measures (anthropometry, lung function, handgrip strength, seated flexibility) in 297 older adults over a period of 3 years (the physical exercise in older people’s lives (PEOPL) study). |
| --- |

12. **Participants**

Describe the number of participants and important characteristics (such as age, gender, location, affiliation, etc.)

| 60 male and female healthy older adults between the ages of 60-74 will be studied after giving their informed oral and written consent. Exclusion criteria are indicated in the Participant Screening Form and Omega-3 Questionnaire. Physician approval is required. |
| --- |

13. **Recruitment**

1. Describe how and from what sources the participants will be recruited, including any relationship between the investigator(s) and participant(s) (e.g., instructor-student; manager-employee).

***Note: Attach a copy of any poster(s), advertisement(s) or letter(s) to be used for recruitment.***

| Participants will be recruited with posters placed in public areas within the city of Guelph community, and the University of Guelph community (see Recruitment Poster). We will also recruit participants by advertising on local radio stations (ie. CJOY). |
| --- |

1. How and where will you contact these participants?

| Interested individuals will contact Samantha Logan by phone or email, and come to the Department of Human Health and Nutritional Sciences in the ANNU building to discuss requirements of the study and their potential involvement. All participants are free to ask any and all questions, and are encouraged to raise any of their concerns. |
| --- |

1. Time required of participants: 10 hour(s) on 8 occasion(s),

| There will be 8 visits allotted for testing. Six of the visits will take ~1 hr, except 2 visits which will take ~2 hrs. The total time commitment will be 10 hours. |
| --- |

**Yes No**

1. Are participants proficient in the language in which the survey is being conducted? X

If not, is translation available?

|  |
| --- |

14. **Compensation**

1. Will participants receive compensation for participation? **Yes No**
   1. Financial  X
   2. Non-financial  X

1. If **Yes** to **either** i) or ii) above, please provide details.

| Participants will pay for the cost of signed medical consent but will receive money for parking prior to each visit. Upon completion of the study participants will receive $100 in cash and a copy of all of their results (please see Appendix E for Participant Results form). In addition, a copy of the completed publication will be available to all participants. This is consistent with other studies in the department involving similar procedures and time commitments. |
| --- |

1. If participants choose to withdraw, how will you deal with compensation?

| Participants will receive compensation for the amount of completed visits. Each visit is worth $12.50 ($100/8 visits= $12.50). The participant will also receive a copy of their current completed results. |
| --- |

**SECTION C – DESCRIPTION OF THE RISKS AND BENEFITS OF THE PROPOSED RESEARCH**

15. **Possible Risks**

- 1. Indicate if the participants might experience any of the following risks: **Yes No**
     1. Physical risk (including any bodily contact or administration of any

substance)? X

- - 1. Psychological risks (including feeling demeaned, embarrassed worried

or upset)? X

- - 1. Social risks (including possible loss of status, privacy and/or reputation)? X
    2. Is there any deception involved?  X
    3. Are any possible risks to participants greater than those the participants

might encounter in their everyday life? X

- 1. If you answered **Yes** to any of points i) through v) above, please explain the risk.

| Participants will have blood samples taken during the study. Participants will also be taking fish oil or placebo (omega 6) oil supplement. Minor side effects may occur in some participants with fish oil supplementation, including belching, and bad breath. In rare cases, side effects may include heartburn, nausea, loose stools, rash, and nosebleeds. These minor side effects can be minimized if the supplement is kept frozen and taken with meals. The investigators will ask the participant if they experienced any adverse effects and will instruct the participant to contact if any adverse effects, such as those listed above, occur. The adverse events will be reported in the event form (See Appendix E Event Form) and this data will be communicated in the final manuscript. There are social risks in this study which will be safeguarded by ensuring that the participant’s information is kept confidential. The participant will be identified by a participant ID number. All documents will be kept in strict confidence, raw data will be stored in a locked cabinet, and electronic data will be encrypted. |
| --- |

- 1. Describe how the risks will be managed (including an explanation as to why alternative approaches could not be used).

| Due to previously outlined objectives of the study, blood sampling is essential. This data cannot be generated by any other means. The side effects of omega-3 fatty acid supplementation can be minimized if the supplements are kept frozen or taken with meals.  Confidentiality is essential in this study. To ensure this, the participant will be identified by a participant ID number. All documents will be kept in strict confidence, raw data will be stored in a locked cabinet, and electronic data will be encrypted. |
| --- |

16. **Possible Benefits**

Discuss any potential direct benefits to the participants from their involvement in the project. Comment on the (potential) benefits to the scientific community/ society that would justify involvement of participants in this study.

| Direct benefits to participants from engagement in this study may potentially benefit the fish oil group to a greater extent than the placebo group. The potential benefits of fish oils include a decrease in resting heart rate and blood pressure; a healthier blood profile; an increase in resting metabolic rate; a greater reliance on fat oxidation for energy both at rest and during exercise; a decrease in fat mass, an increase in lean mass, and greater strength and physical function. For the placebo group we proposed that we will see no significant change from baseline measures. However, beneficial to both groups will be feedback on their own physical measures which will provide each participant with how their values compare relative to clinical healthy guideline values published in scientific literature. The participant will also be involved first-hand research experience to answer the question on whether fish oil supplementation improves various measures of health in community-dwelling adults. This project aids the research community by providing valuable information on the health effects of supplementation with fish oils, since this is the first study of this nature to be conducted. |
| --- |

**SECTION D – THE INFORMED CONSENT PROCESS**

17. **The Consent Process**

1. Describe the process that the investigator(s) will be using to obtain informed consent, including a description of who will be obtaining the informed consent. If there will be no written consent form, explain why.

| Both oral and written consent will be obtained prior to inclusion in the study. The project director, Samantha Logan, will obtain initial oral and written consent. |
| --- |

For information about the required elements in the letter of information and the consent form, please refer to “Instructions for the Preparing Information and Consent Letters” and the sample consent form available at <http://www.uoguelph.ca/research/forms_policies_procedures/human_participants.shtml>.

***Note: Attach a copy of the Letter of Information (if applicable), the Consent Form (if applicable), the content of any telephone script (if applicable) and any other material which will be used in the informed consent process. If the document will be made public, please ensure that it is on University of Guelph letterhead.***

| See attached Participant Consent Form. |
| --- |

1. Will the information provided to the participants be complete and accurate? **Yes** X **No**

If no, please describe the nature and extent of the deception involved. Include how and when the deception will be revealed, and describe the specialized training of the person who will administer this feedback. It is recommended that participants have the opportunity to sign a second consent form, following debriefing when the deception is revealed, to ensure a fully informed consent.

***Note: Attach a copy of the debriefing feedback and, if necessary, a copy of the second consent form on University of Guelph letterhead.***

| Feedback will be given to the volunteer to clear up any deception and provide the volunteer information about their particular physical measures (see section 20). |
| --- |

18. **Consent by an authorized party**

If the participants are minors or for other reasons are not competent to consent, describe the proposed alternate source of consent, including any permission / information letter to be provided to the person(s) providing the alternate consent.

| Not Applicable. |
| --- |

19. **Alternatives to prior individual consent**

If obtaining individual participant consent prior to starting the research project is not appropriate for this research, please explain and provide details for a proposed alternative consent process.

| Not Applicable. |
| --- |

20. **Participant feedback**

Explain what feedback/ information will be provided to the participants after participation in the project. (For example, a more complete description of the purpose of the research, or access to the results of the research).

***Note: Please provide a copy of the written information, if applicable.***

| Upon the completion and analysis of this study we will provide the participants with a copy of their results and the interpretation of the results (See Appendix E for Participant Results form). We will also reveal the participant’s supplement group to the participant. A copy of the manuscript will also be available to all participants. |
| --- |

21. **Participant withdrawal**

1. Describe how the participants will be informed of their right to withdraw from the project. Outline the procedures that will be followed to allow the participants to exercise this right.

| Participants will be informed prior to the start of each testing session of their right to withdraw, and ensured that they should not feel pressured to continue. Participants can withdraw at any time during the study, by informing any of the investigators of their desire to do so. Participants will be required to return any unused supplements to the investigator. Any participant that withdraws will be replaced, and recruited in the same manner as all other subjects. At any point during the study, participants will be asked to withdraw if they alter their diet or lifestyle, have an adverse reaction to the supplement, or begin taking any medication, supplements, or herbal products listed in the participant screening form. |
| --- |

1. Indicate what will be done with the participant’s data and any consequences for the participant of withdrawing from the study.

| There will not be any consequences for a participant withdrawing from the study. A participant’s data will be withdrawn upon request. |
| --- |

1. If the participants will not have the right to withdraw from the project, please explain.

| Not Applicable. |
| --- |

**SECTION E – CONFIDENTIALITY**

**22. Ensuring confidentiality**

**Yes No**

1. Will all participants be anonymous?  X
2. Will all data be treated as confidential? X

**Please note the difference:** Participants’ identity/data will be confidential if an assigned ID code or number is used, but it will not be anonymous. Anonymous data cannot be traced back to an individual participant.

1. Describe the procedures to be used to ensure anonymity of participants and/or confidentiality of data both during the conduct of the research and in the release of its findings.

| Once collected and tabulated, all data will be identified in a confidential fashion (e.g. Participant1, Participant 2, etc.). Under no circumstances will any reported or published data from this study contain the identity of any of the volunteering participants. Data will be stored in databases on the researchers’ personal computers. These are two laptops (Dr. Spriet’s and Samantha Logan’s), which will be file encrypted to ensure complete confidentiality. Hard copies of data summaries will also be made and kept on file. A master list and all data will be kept for three years in a locked cabinet following completion of the study. |
| --- |

1. Explain how written records, video/audio tapes and questionnaires will be secured, and provide details of their final disposal or storage.

| Data will be stored in databases on the researchers’ personal computers. These are two laptops (Dr. Spriet’s and Samantha Logan’s), will be file encrypted to ensure complete confidentiality. Hard copies of data summaries will also be made and kept on file in a locked cabinet. All data will be encoded by participant identification numbers in order to ensure confidentiality. A master list and all data will be kept for three years in a locked cabinet following completion of the study. |
| --- |

1. If participant anonymity or confidentiality is not appropriate to this research project, explain, providing details of how all participants will be advised of the fact that data will not be anonymous or confidential.

| Not Applicable. |
| --- |

**SECTION F – MONITORING ONGOING RESEARCH**

23. **Annual Review and Adverse Events**

1. Minimum protocol review requires the completion of a “Renewal/Completed Status Report” at least annually. Indicate whether any additional monitoring or review would be appropriate for this project.

***Note: It is the investigator’s responsibility to notify the REB using the “Renewal/Completed Status Report” when the project is completed, or if it is cancelled.*** ***The form is available at*** <http://www.uoguelph.ca/research/forms_policies_procedures/human_participants.shtml>***.***

| Additional monitoring would not be appropriate with respect to this project. |
| --- |

1. **Adverse events** (unanticipated negative consequences or results affecting participants) must be reported to the Research Ethics Board and the Research Ethics Coordinator as soon as possible.

24. **Additional Information**

(Use an additional page if more space is required to complete any sections of the form, or if there is any other information relevant to the project that you wish to provide to the Research Ethics Board.)

| Please see attached supplement information (Appendix E). |
| --- |

**SECTION G – SIGNATURES**

**Responsible Faculty Assurance:**

**I­­, ____________________________ [PLEASE PRINT] have the ultimate responsibility for the conduct of the study described in this application including my responsibilities as an advisor to any students involved in this project. I have read and am responsible for the content of this application. If any changes are made in the above arrangements of procedures, or adverse events are observed, I will bring these to the attention of the Research Ethics Coordinator.**

      (yyyy-mm-dd)

**Signature Date**
